# Cryo-EM analysis of homodimeric full-length LRRK2 and LRRK1 protein complexes

**DOI:** 10.1038/s41598-017-09126-z

**Published:** 2017-08-17

**Authors:** Kushal Sejwal, Mohamed Chami, Hervé Rémigy, Renée Vancraenenbroeck, William Sibran, Rosmarie Sütterlin, Paul Baumgartner, Robert McLeod, Marie-Christine Chartier-Harlin, Veerle Baekelandt, Henning Stahlberg, Jean-Marc Taymans

**Affiliations:** 1 Center for Cellular Imaging and NanoAnalytics (C-CINA), Biozentrum, University of Basel, Basel, 4056 Switzerland; 2 FEI Company, Eindhoven, The Netherlands; 3 KU Leuven, Laboratory for Neurobiology and Gene Therapy, Department of Neurosciences, 3000 Leuven, Belgium; 4 Université de Lille, Inserm, CHU Lille, UMR-S1172 - JPArc - Centre de Recherche Jean-Pierre AUBERT Neurosciences et Cancer, F-59000 Lille, France; 5 Department of Structural Biology, Weizmann Institute of Science, Rehovot, 76100 Israel

## Abstract

Leucine-rich repeat kinase 2 (LRRK2) is a large multidomain protein implicated in the pathogenesis of both familial and sporadic Parkinson’s disease (PD), and currently one of the most promising therapeutic targets for drug design in Parkinson’s disease. In contrast, LRRK1, the closest homologue to LRRK2, does not play any role in PD. Here, we use cryo-electron microscopy (cryo-EM) and single particle analysis to gain structural insight into the full-length dimeric structures of LRRK2 and LRRK1. Differential scanning fluorimetry-based screening of purification buffers showed that elution of the purified LRRK2 protein in a high pH buffer is beneficial in obtaining high quality cryo-EM images. Next, analysis of the 3D maps generated from the cryo-EM data show 16 and 25 Å resolution structures of full length LRRK2 and LRRK1, respectively, revealing the overall shape of the dimers with two-fold symmetric orientations of the protomers that is closely similar between the two proteins. These results suggest that dimerization mechanisms of both LRRKs are closely related and hence that specificities in functions of each LRRK are likely derived from LRRK2 and LRRK1’s other biochemical functions. To our knowledge, this study is the first to provide 3D structural insights in LRRK2 and LRRK1 dimers in parallel.

## Introduction

Parkinson’s disease (PD) is the second most common neurodegenerative movement disorder. It affects 1–2% of all people above the age of 65^1^ and is at present incurable, although treatments are available to alleviate the symptoms. Genetic studies have identified several genes involved in PD pathogenesis. The leucine rich repeat kinase 2 (*LRRK2*) gene is of particular importance, with mutations in the coding sequence being the most prevalent known causes of genetic PD and genomic variants at the LRRK2 locus being common risk factors for sporadic PD^2^. In addition, LRRK2 appears to act upstream of several other PD genes and PD risk factors, such as alpha-synuclein, tau, cyclin G associated kinase (GAK) and RAB7L1^3, 4^. The 144 kb-long LRRK2 gene encodes for the 2527 amino acids long, cytosolic enzyme LRRK2, which functions as a GTPase as well as a kinase. Most of the pathologically important mutations are clustered in the catalytic core of this protein, hinting that altered GTPase and kinase activities may play a crucial role in pathogenesis^5, 6^. Targeting the LRRK2 signaling pathway is currently regarded as one of the most promising approaches in drug development for PD^7–10^.

**Figure 1:**
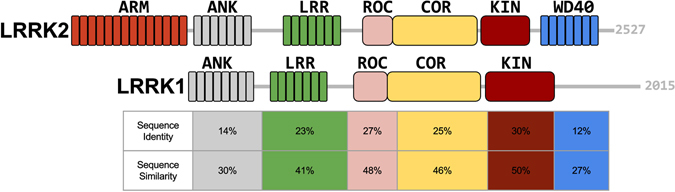
Domain organisation of LRRK2 and LRRK1. LRRK2 and LRRK1 domains are depicted with different colors and their relative location is drawn to scale within the full-length protein: ARM, armadillo repeats; ANK, ankyrin repeats; LRR, leucine-rich repeats; ROC, Ras Of Complex proteins GTPase; COR, C-terminal Of ROC; KIN, a kinase; WD40, WD40 repeats. The sequence identity and sequence similarity is reported in the column with same color below each domain (adapted from ref. 20). A detailed alignment between the human LRRK2 and LRRK1 sequences for each homologous domain is given in the supplemental data.

LRRK2 is a member of the ROCO protein family^11^. It contains several protein-protein interaction domains, including armadillo (ARM), ankyrin repeats (ANK), leucine-rich repeats (LRR), Ras Of Complex proteins GTPase (ROC), C-terminal Of ROC (COR), a kinase (KIN) and WD40^12^ (Fig. 1). The multidomain protein is involved in several cellular functions, including autophagy and neurite outgrowth regulation, and is related to some mitochondrial diseases^13–15^. Biochemical experiments suggest that the kinase and GTPase activities of LRRK2 are regulated by dimerization^16–19^.

Three other ROCO proteins have been identified in humans: Leucine-rich repeat kinase 1 (LRRK1), death-associated kinase 1 (DAPK1), and malignant fibrous histiocytoma amplified sequence 1 (MFASHI1). LRRK1 is the closest homologue of LRRK2. The domain organization is similar and, like LRRK2, LRRK1 is known to purify as homodimer^20^. LRRK1 (2015 amino-acids long) lacks the ARM domain present in LRRK2 and, although a C-terminal kinase region is present, has no WD40 domain. Otherwise, the sequence identity and similarity between the domains of the two proteins varies between 14% and 50% (Fig. 1, and supplemental data). Despite this similarity, mutations in LRRK1 have not been linked to PD. This difference has stimulated various studies comparing the functional roles of LRRK1 and LRRK2^21–23^.

Much available structural knowledge about LRRK2 comes from the study of related ROCO proteins from lower organisms. So far, crystal structures have been published for the ROC (PDB ID code 3DPT) and ROC-COR (PDB ID code 3DPU) domains of the ROCO protein of the thermophilic bacterium *Chlorobium tepidum* and the kinase domain (PDB ID code 4F0F) of the ROCO4 protein of the slime mold *Dictyostelium discoideum* ^24–26^. Although, these structures have advanced our understanding of LRRK2 functions, the inferred functionality of the full-length protein is indirect. The structural properties of individual human LRRK2 domains have also been studied, including the ROC (PDB ID code 2ZEJ)^24^ and the LRR^27^ domains. Recently, a low-resolution structure of the 3-flag tagged wild-type LRRK2 dimer was obtained by negative stain transmission electron microscopy (TEM), indicating a compact architecture^28^. Structural information on LRRK1 is minimal; no 3D structures have been reported, for the full-length protein or fragments thereof.

In this study, we report a buffer optimization for LRRK2 and LRRK1 protein production, using ProteoPlex. ProteoPlex is a screening method based on the Thermofluor^®^ differential scanning fluorimetry assay and can be used to assess the stability of multidomain macromolecular protein complexes in a given buffer system. ProteoPlex uses sparse-matrix screening of a protein’s thermal unfolding behavior under various conditions to find the optimum buffer conditions favoring stable and monodisperse complexes^29^. Similar methods based on differential scanning fluorimetry (DSF) are routinely employed in X-ray crystallography to optimize the quality and quantity of protein samples for further crystal screening^30^, but so far have rarely been employed in combination with cryo-electron microscopy (cryo-EM) studies.

In this study, we also report the 16 Å and 25 Å resolution 3D structures of the homodimeric full-length 3xflag-LRRK2 and 3xflag-LRRK1 complexes respectively, determined by cryo-EM. The use of TEM to evaluate protein quality and buffer optimization using differential scanning fluorimetry enabled the optimization of the LRRK protein purifications for the cryo-EM analysis. Both, the 2D analysis of LRRK2 and LRRK1 images, and their 3D models, reveal a striking similarity between the tertiary and quaternary structure of the two protein dimers.

## Results

### Detergent is required for LRRK2 to remain correctly folded

The production of protein in high yield and purity is a prerequisite for structural analysis by cryo-EM^31, 32^. Pooling the cell lysate from several Petri dishes and eluting the protein in less volume (see *Materials and Methods*) resulted in protein concentrations as high as 1 mg/ml for both LRRK2 and LRRK1, regardless of the buffer system used (see below). This concentration was sufficient for initial negative stain TEM imaging, and for cryo-EM if the Quantifoil grids employed were coated with an additional carbon film. Ultra-centrifugal filter devices could not be used to concentrate either protein, because both tended to precipitate and bind to the filter membrane (data not shown).

**Figure 2:**
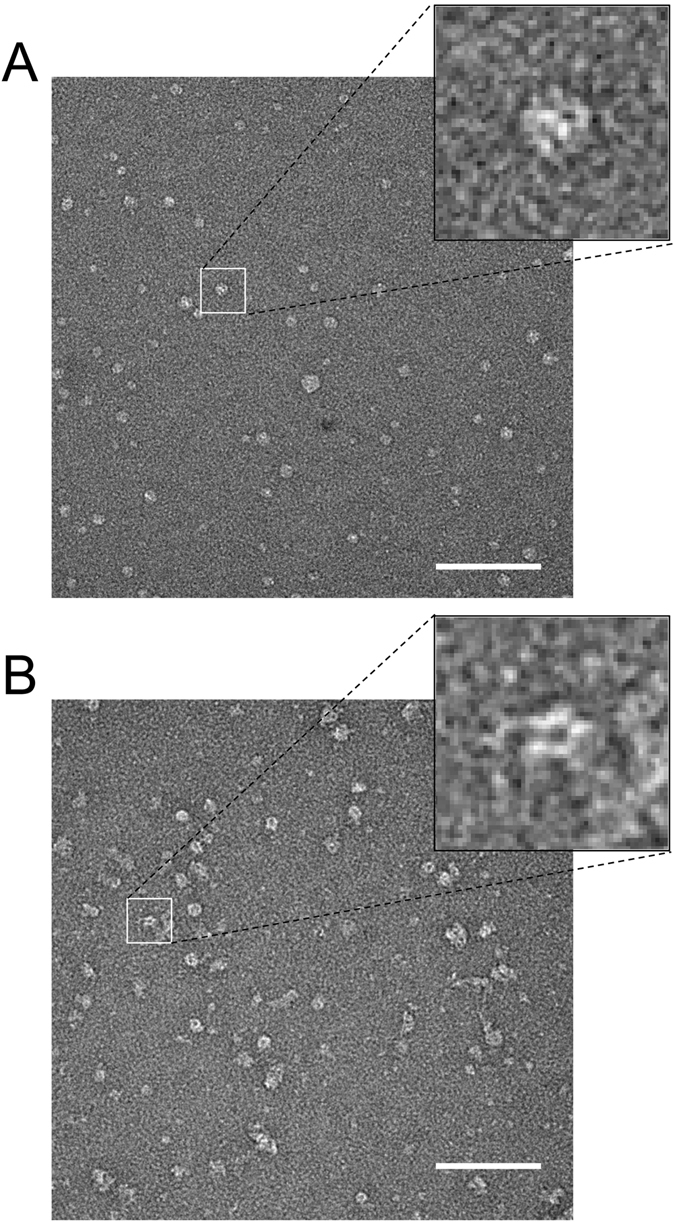
Effect of detergent on the 3xflag-LRRK2 structure. Negative stain TEM images of LRRK2 eluted using in 20 mM Tris/HCl pH 7.4, 200 mM NaCl, 5 mM MgCl_2_, 1 mM DTT. (**A**) Without the addition of detergent; almost all of the particles have a globular appearance and structural details are not visible. (**B**) With 0.02% of Triton X-100; many of the particles have an elongated shape and some structural detail is evident. Dimeric sides-views are clearly visible (inset). However, there are also some globular particles demonstrating that even in the presence of detergent the protein is not completely stable when the Tris buffer is employed (see *Materials and Methods* and Fig. 4). Scale bars: 100 nm. Insets are 5 times magnified.

We evaluated the possibility to perform EM analysis of recombinant LRRK2 in the absence of detergent. For this, purifications of 3xflag-LRRK2 were carried out as detailed in Section 2.1.3, including lysis and wash steps with buffers containing detergent, but with omission of detergent in the final rinse and elution steps. Under these conditions, negative stain TEM of the protein preparations revealed globular particles of various sizes that had no recognizable structure (Fig. 2A). In contrast, a small population of elongated particles with features was observed when 0.02% (v/v) Triton X-100 was included in the elution buffer (Fig. 2B). Protein stability did not increase further when other nonionic detergents were tested; the purity and quality of the eluted protein remained the same, when Triton X-100 was replaced by 0.02% (v/v) n-decyl-β-D-maltopyranoside (DM), NP-30, lauryl maltose neopentyl glycol (LMNG) or Tween 20 (data not shown). Consequently, 0.02% (v/v) Triton X-100 was included in all buffers used to elute 3xflag-LRRK2 and 3xflag-LRRK1 for EM experiments.

**Figure 3:**
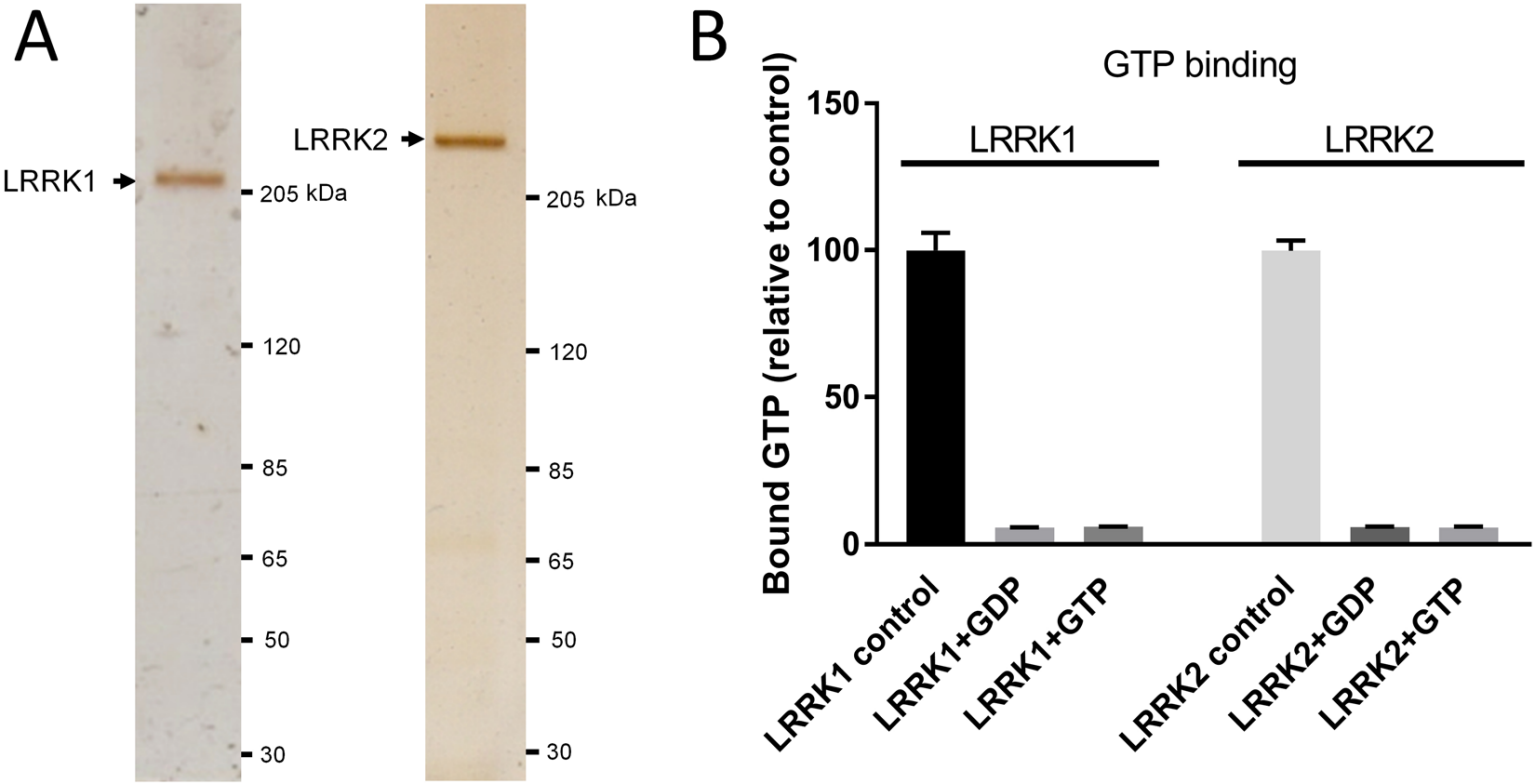
Expression and purification of full-length 3xflag-LRRK2 and 3xflag-LRRK1. (**A**) Silver stained gel of 3xflag-LRRK2 and 3xflag-LRRK1 purified as described in *Materials and Methods*. In both cases, the protein concentration was 10 μg/ml and a 10 μl aliquot was added to the gel. The dominant bands observed at the molecular weights 215 and 285 kDa are 3xflag-LRRK2 and 3xflag-LRRK1, respectively, indicating highly pure protein fractions. Markers are in kiloDaltons. (**B**) Functionality of the purified 3xFlag-LRRK1 and 3xFlag-LRRK2 proteins was assessed using a radiometric GTP binding assay as described in materials and methods. Proteins were incubated at 30 °C with GTP-α-P32 alone or with addition of 200 mM ‘cold’ nucleotides. After incubation, excess nucleotides were rinsed away and the amount of bound isotopic GTP was measured via scintillation counting and expressed as binding level relative to control protein (n = 3).

**Figure 4:**
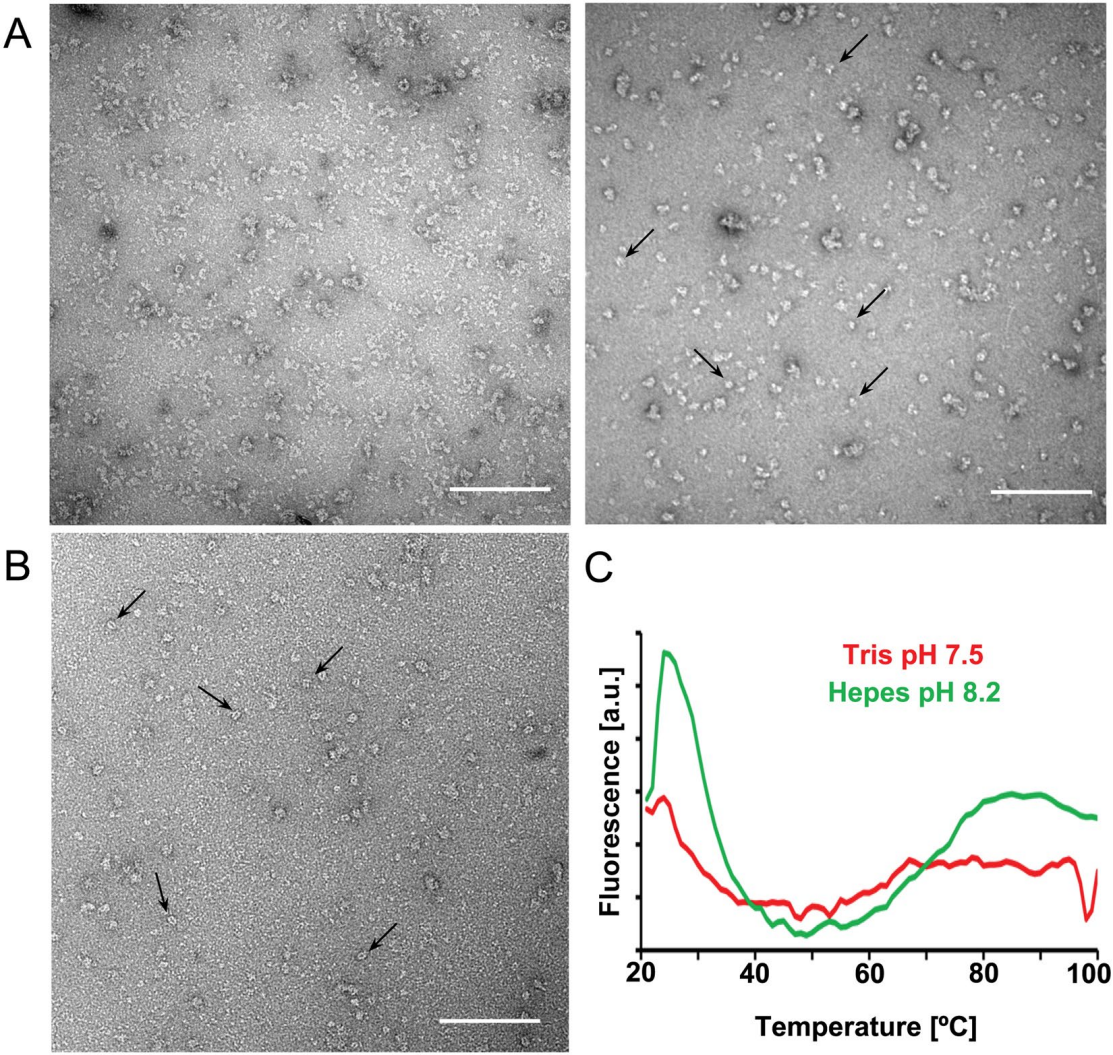
Negative stain TEM of 3xflag-LRRK2 and 3xflag-LRRK1 and buffer optimization for 3xflag-LRRK2 by ProteoPlex screening. (**A**) (Left) Negative stain TEM micrograph of LRRK2 purified in binding buffer at pH 7.5 and eluted in elution buffer Tris/HCl at pH 7.4 after a wash step. The sample is heterogeneous with many disintegrated protein particles in the background and some aggregates. (Right) Negative stain TEM micrograph of the LRRK2 sample purified in binding buffer at pH 6.8 and eluted in elution buffer at pH 8.2 as suggested by ProteoPlex screening. Although there is still some heterogeneity, more particles are intact (arrows) and the background is much cleaner. Scale bars: 100 nm. (**B**) Negative stain TEM micrograph of LRRK1 purified as described in *Materials and Methods* with Tris/HCl based binding buffer at pH 7.5 and eluted in Tris/HCl based elution buffer at pH 7.4. Distinct dimeric LRRK1 particles can be clearly seen (arrows) with few aggregates and smaller particles in the background. Scale bars: 100 nm. (**C**) ProteoPlex melting curve profiles obtained for LRRK2 eluted in Tris pH7.4 and Hepes pH8.2. Relative fluorescent intensity is plotted for the temperature range of 20°–100 °C. The standard ProteoPlex algorithm could not be used to calculate a thermodynamic model, as the fluorescence signal was too low. Trend lines were calculated using a moving average with a period of 2. The melting curve displays already aggregation at 20 °C when a Tris pH 7.4 buffer was used to elute LRRK2, whereas a peak occurs at 25 °C and the curves displays a 2-state unfolding between 20 and 40 °C when the elution was performed with Hepes 8.2, indicating that this buffer places the LRRK2 protein in more favorable conditions, *i*.*e*., predictive of stable and monodisperse protein complexes. This latter buffer was then used as the basis for LRRK2 elution in subsequent preparations of LRRK2 protein for cryo-EM. Additional melting curve profiles for the buffer screens (binding buffer and elution buffer screens) are given in Supplementary Figures S1, S2 and S3.

Silver stain gels showed that the purified LRRK2 and LRRK1 samples were pure (Fig. 3). However, images of purified LRRK2 showed a very heterogeneous mixture, even though detergent was present (Fig. 4A, left panel). Compared to LRRK2, negative stain TEM images of pure LRRK1 were slightly less heterogeneous and revealed a large population of similarly sized particles (Fig. 4B). This prompted us to perform buffer optimization experiments, in order to identify buffers that reduce particle heterogeneity.

### Buffer optimization by ProteoPlex screening

Three ProteoPlex assays^29^ were performed to define (i) the optimal buffer conditions and (ii) the effect of buffer ligands/additives for the stability of affinity-bound 3xflag-LRRK2 protein, which correlates to the optimal lysis/binding buffer for the purification and (iii) the optimal buffer conditions for the elution of affinity-bound 3xflag-LRRK2. For assay (i), purified protein loaded to affinity resin was prepared and submitted to ProteoPlex analysis as described in *Materials and Methods*. In assay (i), substantial peaks indicating protein unfolding or aggregation were detected with many buffers in the temperature range between 20 and 40 °C. Hepes at pH 6.8 proved to be the most favorable buffer (Supplementary Figure S1). In assay (ii), subsequent screening of 88 different buffer ligands/additives with 3xflag-LRRK2 purified with lysis, wash and rinse buffers in which Tris pH 7.5 was replaced by Hepes at pH 6.8 and still bound to the affinity beads. Screen (ii) showed that CaCl_2_, MgCl_2_, NH_4_Cl, gluthathion (oxidized and reduced), ethylene glycol, trehalose, and glycerol had a stabilizing effect on the protein (Supplementary Figure S2). As a result, subsequent purifications were carried out using the adapted buffer system, 20 mM Hepes pH 6.8, 10 mM CaCl_2_, 5 mM MgCl_2_, 100 mM NH_4_Cl, 1% (v/v) Triton X-100, 5% (v/v) glycerol. Protein purified with this buffer for the lysis and wash steps was used in assay (iii) performed to find the best elution conditions. As described in *Materials and Methods*, the affinity bound protein was first rinsed in test elution buffers (effectively removing Triton X-100 and glycerol). In this elution buffer screen, Hepes at pH 8.2 was the most favorable buffer (Supplementary Figure S3). Therefore, we adapted the elution buffer composition for 3xflag-LRRK2 to 20 mM Hepes pH 8.2, 100 mM NH_4_Cl, 5 mM MgCl_2_, 10 mM CaCl_2_, and 0.02% Triton X-100.

The overall result of the ProteoPlex assays is summarized in Fig. 4C, where the melting curves of 3xflag-LRRK2 in the initial (based on Tris pH 7.5, see *Materials and Methods*) and adapted buffer systems (based in Hepes 6.8 in the lysis and wash buffers and Hepes 8.2 in the elution buffer, see above) are superimposed showing the clear two-state melting curve for the adapted condition. Although, the yield of a new LRRK2 purification carried out using the adapted buffers, HEPES buffer at pH 6.8, for cell lysis and protein extraction by affinity binding, and HEPES buffer at pH 8.2 to elute it from the affinity column, was comparable to the yield of control purifications carried out under the initial conditions with Tris/HCl buffers, the sample was of higher quality. Indeed, negative stain TEM images revealed a larger population of intact complexes, and the background was much cleaner with fewer aggregates (Fig. 4A, right). These adapted purification conditions were, therefore used to purify 3xflag-LRRK2 for cryo-EM analysis.

### Cryo-EM of LRRK2

3xflag-LRRK2 protein purified with optimized lysis, wash and elution buffers (see above) was applied to glow-discharged thin (5 nm) carbon films supported by holy carbon Quantifoil grids, vitrified and imaged as described in *Materials and Methods*. A thin carbon support film was essential, even though it can influence protein orientation, add additional noise and impair CTF correction. In its absence, proteins were only present on the thick carbon mesh, none were found in the vitreous ice spanning the holes; neither increasing the protein concentration tenfold, nor using Lacey grids, which have mesh-like openings of different sizes and shapes, helped (Supplementary Figure S4).

**Figure 5:**
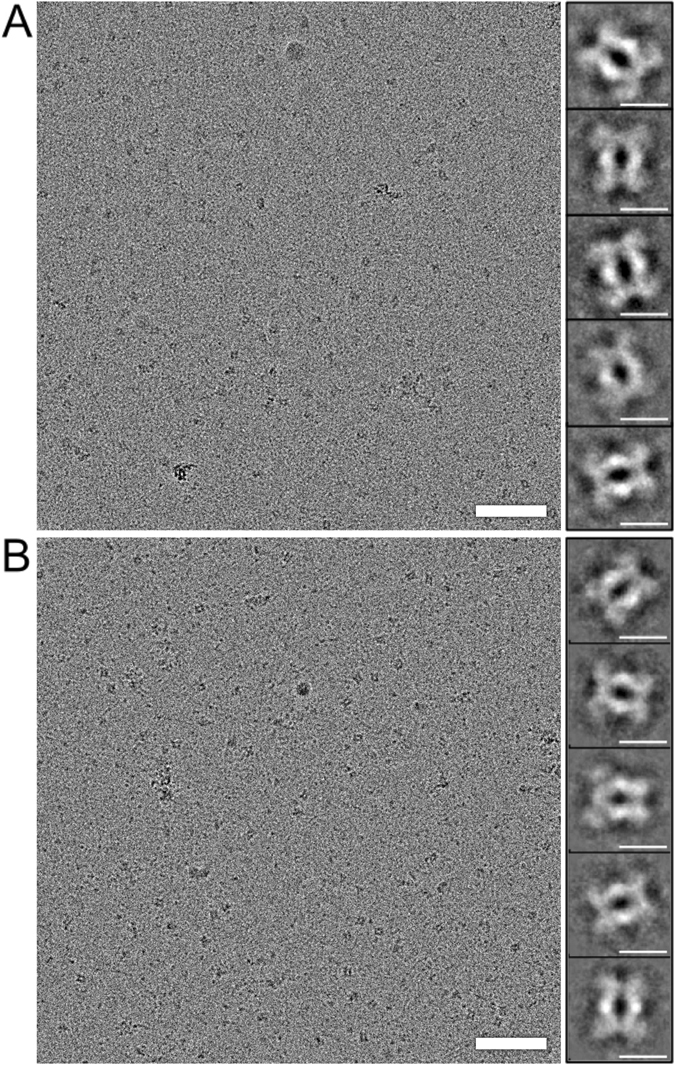
Cryo-EM and single particle processing of 3xflag-LRRK2 and 3xflag-LRRK1. (**A**) LRRK2 complexes eluted in Hepes buffer at pH 8.2, imaged by cryo-EM. Right: Selected EMAN2 class averages showing characteristic side-views of the complex. Particles in this view are ~17 nm long and ~12 nm wide. Their two-fold symmetry indicates that the complex is a dimer. (**B**) LRRK1 complexes eluted in Tris buffer at pH 7.4, imaged by cryo-EM. Right: Selected EMAN2 class averages showing characteristic side-views of the LRRK1 complex. These are very similar to corresponding side-views of LRRK2 but slightly smaller, being ~16 nm long and ~10 nm wide. The dimeric arrangement is apparent. Scale bars: 100 nm for the micrographs, 10 nm for the averages.

Elongated particles with two-fold symmetry could be distinguished in the raw micrographs of 3-flag-LRRK2 cryo-EM preparations (Fig. 5A). To obtain a 3D model, 15′352 particles were selected from the 279 micrographs recorded and subjected to reference free alignment and classification as detailed in the *Materials and Methods*. The 128 class averages generated (Supplementary Figure S5A) show that the LRRK2 complexes were randomly oriented on the grid and reveal a high degree of flexibility; preferential orientation due to the presence of a continuous carbon film was not detected. Side-views of the particles are very characteristic, being ~17 nm-long, ~10 nm-wide with a clear 2-fold symmetry, suggesting that the complex is a dimer (Fig. 5A). The dimensions are similar to previous estimates^20^. The two 3-flag-LRRK2 monomers interact at both ends of their protein cores, which are aligned in parallel and seem to form a small cavity. The ends of the complex are less distinct, indicating that these domains have intrinsic flexibility. To validate the analysis, the same dataset was also processed with the RELION software package^33^, which uses a Bayesian approach to infer the parameters of a statistical model from the data. The resulting class averages are very similar to those obtained using EMAN2 (Supplementary Figure S5B).

### Cryo-EM of LRRK1

3xflag-LRRK1 protein purified as described in *Materials and Methods* was analyzed according to the same procedure. To obtain a 3D model, 5′363 particles were selected from 55 micrographs, aligned without the use of a reference and classified into 64 classes containing a minimum of 100 particles each, using EMAN2 (Fig. 5B). Due to the lower number of particles per class, the averages obtained look less crisp as their LRRK2 counterparts, but the characteristic side-views are clearly visible (Fig. 5B). These side views show that LRRK1 forms a dimer that is very similar to the LRRK2 dimer, but slightly smaller. As might be expected from the two sequences (Fig. 1), the protrusions at the ends of the LRRK1 complex are less prominent.

### 3D Reconstruction of LRRK2 and LRRK1

The initial 3D models generated for both LRRK2 and LRRK1 using spacegroups C1 (no symmetry) and C2 (two-fold symmetry), respectively, were in good agreement, which was also evident from the dimeric shape of the 2D class averages (Fig. 5). Using the respective initial model as reference, iterative projection-matching reconstruction was applied without imposing any symmetry. After three rounds of refinement, the final reconstruction was produced at a resolution of 24.2 Å and 24.3 Å for 3xflag-LRRK2 and 3xflag-LRRK1, respectively (Supplementary Figure S6).

**Figure 6:**
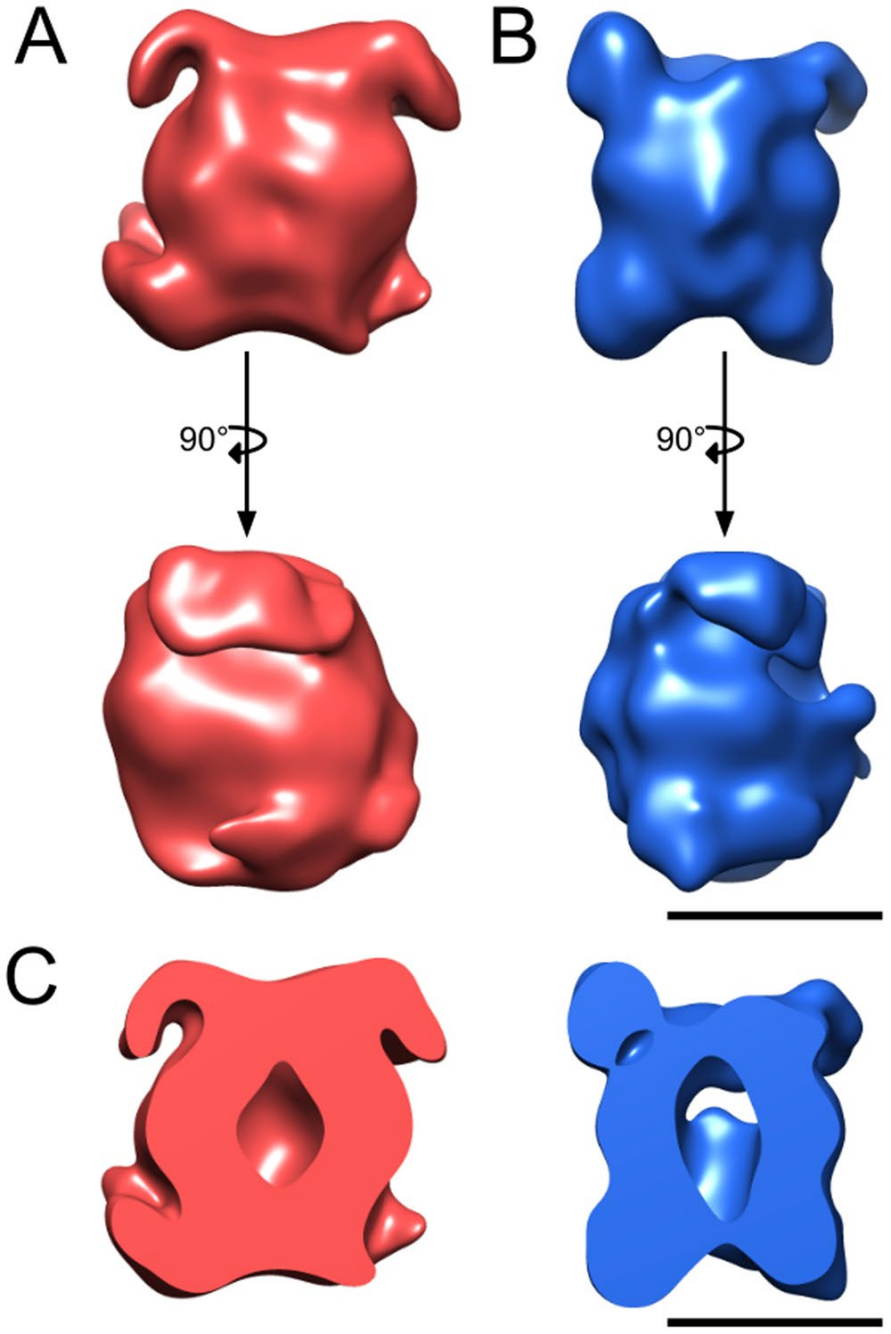
3D reconstructions of the homodimeric 3xflag-LRRK2 and 3xflag-LRRK1 complexes. (**A**) 3D model of 3xflag-LRRK2 (red) at 24.2 Å resolution showing the side-view orientations, the lower one being 90 degrees rotated relative to the upper one. (**B**) 3D model of 3xflag-LRRK1 (blue) at 24.3 Å resolution showing two side-view orientations, the lower one being 90 degrees rotated relative to the upper one. (**C**) Cut-away of 3xflag-LRRK2 (red) and 3xflag-LRRK1 (blue). The plane of the slice is perpendicular to the presumed dimer axis. Scale bars: 10 nm.

**Figure 7:**
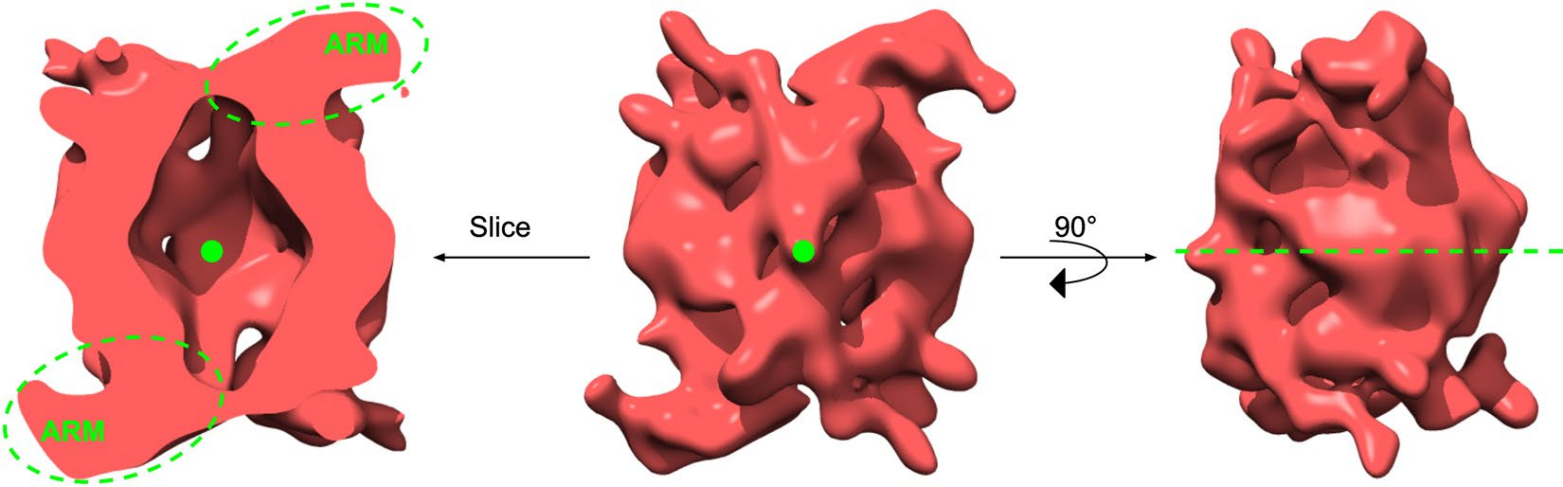
3D reconstruction of the homodimeric LRRK2. 3D model of LRRK2 at 16.2 Å resolution showing the side-view orientations, the right one being 90 degrees rotated relative to the center one. The left is a cut-away of LRRK2 with the plane of the slice. Also shown are the dimerisation axis between the LRRK2 monomers (green dot and dashed green line). In the left and the central panel, the dimerization axis points towards the reader. In the right panel, it points form left to right along the middle of the molecule. The label for N-Terminal armadillo repeats domain, ARM are depicted in the left panel. The ARM domain here is visible only in the front, due to the perspective display that hides the rear ARM domain behind the molecule.

The overall shape of the LRRK2 and LRRK1 complexes is similar (Fig. 6). In both cases, the central regions of the two monomers form an elliptical homodimer with what appears to be a central cavity (Fig. 6C). However, as this region of the monomer is slightly more curved for the LRRK2 dimer, the cavity is smaller. Two dimerization contacts are visible in both low-resolution maps. These contacts are at roughly equal distances from the center of the complex, towards the N and the C terminal, respectively. At this resolution, it was not possible to assign and fit domains to the EM density. However, the two-fold symmetric arrangement of the two monomers is evident in the non-symmetrized reconstructions, and the symmetry axis of the C2 symmetric complex is in the direction of the longer axis of the molecule, which is vertical in Figs 6 and 7. This symmetry axis is also apparent in the 2D class averages, shown in Fig. 5. The 3D reconstructions also show the central protein region, which is common to both LRRK2 and LRRK1, to form a small cavity in the dimer. We tentatively interpret this region as the catalytic core of the complex comprised of the ROC-COR-kinase domains found in both proteins.

**Figure 8:**
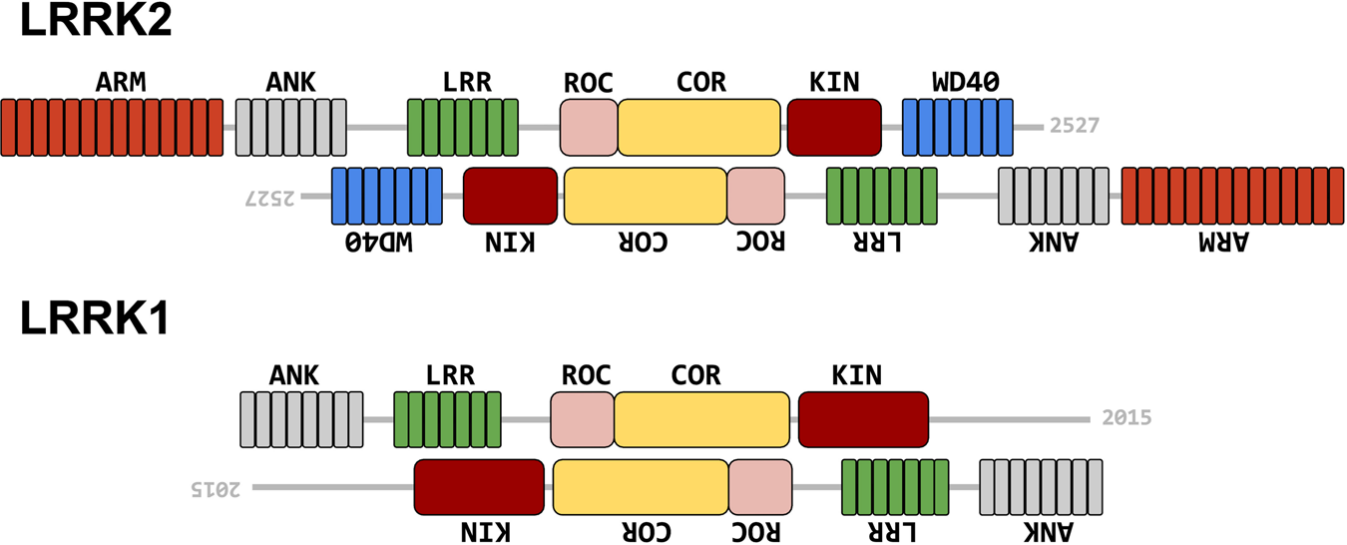
A model for the antiparallel arrangement of protomers in the LRRK2 and LRRK1 dimers. Depicted are the proposed overall orientations of the LRRK2 and LRRK1 dimers as derived from the models calculated from cryo-EM experiments, showing antiparallel orientation of LRRK2 and LRRK1 protomers in their respective dimers, as well as the extension of the N-terminal domain outside of the antiparallel dimer.

To increase the resolution and address conformational heterogeneity in the data, a bigger dataset for LRRK2 was recorded by cryo-EM. A total of 279 micrographs of LRRK2 were collected which yielded a total of 15′352 particles, out of which only 8′239 particles were retained after 2D classification in RELION. These particles were used to perform 3D classification in RELION using four classes with C2 symmetry. The resulting 3D structures of four classes were similar with roughly equal number of particles. The best class, which represented 27.2% of the total particles, resulted in an EM density map at 16.2 Å resolution (Fig. 7). The resolution was calculated based on the spectral signal-to-noise ratio curve (SSNR) (Supplementary Figure S7). Via this higher resolution EM density map, the 3D model of LRRK2 revealed an antiparallel arrangement of the LRRK2 protomers in the homodimer as well as information on which domains interact which each other. The extra density protruding from each trans end of the dimer can be attributed to the N-terminal ARM domain while the WD40 domain is likely associated with either ANK or LRR domain to form a dimer. Based on our EM model, we propose a model for the domain arrangement of LRRK2 and LRRK1, showing antiparallel dimers with suggested domains extending beyond the dimer core (Fig. 8).

## Discussion

The determination of the full-length structure of both LRRK2 and LRRK1 is an important goal to further our understanding of the molecular mechanisms underpinning functions of LRRK proteins. Also, given that LRRK2 is a key player in the pathogenesis of Parkinson’s disease while LRRK1 is not, the comparison of structures of both LRRK proteins may yield clues to LRRK2’s pathological functions and aid in designing novel LRRK2 targeting therapeutics. Our study worked on two aspects, the improvement of protein quality for structural analysis and performing single particle analysis cryo-EM. Buffer screening and calculating associated thermal shift assays using ProteoPlex revealed that LRRK2 favors a higher pH to be stably and correctly folded in solution. It should be noted that the pH of most of negative stain used routinely in EM for structural analysis is either acidic or neutral, e.g., uranyl formate is acidic with a pH ~ 4^34^. Although it has been shown that the fixation rate is very rapid and the low pH usually does not affect the overall protein conformations, a possible effect of the acidic stain on the protein state cannot be ruled out completely, especially for pH-sensitive protein complexes. As observed in comparing Figs 4 and 5, the raw LRRK2 and LRRK1 particles in negative stain micrographs displayed a different visual aspect compared to the cryo-EM micrographs, owing to low pH exerted by negative stain on LRRKs.

Taking into account the potential effect of negative staining on the LRRK structures, we used unstained cryo-EM to collect structural data for LRRK2 and LRRK1. The generated 3D maps show full-length 16 and 25 Å resolution structures of LRRK2 and LRRK1, respectively, revealing the overall shape of the dimers that is closely similar between the two proteins. Our study presents the first 3D of the full-length human LRRK1 dimer structure and provides additional structural details to the recent report of the LRRK2 dimer structure based on a negative stain EM^28^. Comparing the structure reported in Guaitoli *et al*.^28^ with the LRRK2 structure presented here, both structures show dimer particles of similar size, and both dimers show tightly packed domains with dimerization occurring over a single twofold rotation axis. Also, both structures are compatible with a model in which the LRRK2 ARM domain extends outside of the LRRK2 dimer core (Figs 6 and 7), a finding corroborated by the low level of cross-linking observed between the ARM domain and other LRRK2 domains^28^. In contrast, the slightly higher resolution LRRK2 structure in our study (16 Å in Fig. 7 vs. 22 Å in ref. 28) reveals additional details, in particular that the homodimer protomers are arranged in two-fold symmetric orientation forming an elongated cavity, and that the ARM domain curves away from the dimer core instead of extending along the dimer axis. As noted above, these discrepancies may be due to the use of a buffer specifically optimized here for structural biology analysis of LRRK2 in solution, the use of the cryo-EM technique that maintains complexes in a 3D matrix rather than 2D matrix for negative stain techniques and/or the improved resolution of the structure obtained in the present study.

At the resolutions obtained here for the LRRK2 and LRRK1 structures, fitting of domains in the EM density maps is not possible. However the arrangement of the protomers in the LRRK2 and LRRK1 dimers can be inferred indirectly. The protomers in both the complexes arrange in a two-fold symmetric orientation where the C-terminal kinase and WD40 domains of one protomer interact with N-terminal ankyrin and LRR domains of the other. The symmetric orientation of the LRRK2 protomers is consistent with other observations including that LRRK2 autophosphorylates itself primarily in the ROC domain. Indeed, the ROC domain of one protomer would in this instance be accessible to the kinase domain of the second protomer, although intramolecular ROC phosphorylation is not excluded^17^. Interestingly, the cavity formed within the dimer provides additional space for flexibility of the LRRK proteins and may play a role in dynamic interactions between functional domains. Another feature of the LRRK2 and LRRK1 models is that large surface areas of the proteins remain available for further interactions. For instance, binding interfaces of specific domains are known to be mapped to specific domains such as p21-activated kinase 6 (PAK6) and tubulin to ROC, heat shock protein 90 (HSP90) to Kinase, or N-ethylmaleimide sensitive fusion (NSF) to WD40^35–38^.

Interestingly, the striking similarity on the overall structures of LRRK2 and LRRK1 suggests that the specificity of LRRK2 versus LRRK1 in its role in Parkinson’s disease must be sought at another level. A first hypothesis is that LRRK2’s specificity is largely mediated by its N-terminal domain that is shown here to be located on the fringe of the LRRK2 dimer core (Figs 6 and 7 and ref. 28). Alternatively, structural subtleties may be present within the respective dimer cores of LRRK2 and LRRK1 responsible for differences in the regulation of its biochemical functions or of its protein-protein interactions. Finally, based on the high structural variability observed here, it should be noted that the LRRK2 and LRRK1 models presented constitute only one of several conformations of these proteins. Several studies have for instance suggested that dimerization may influence the GTPase and kinase functions^39^, indicating that different dimer conformations may lie at the basis of these functional differences.

The work presented here lays the foundations for further exploration into LRRK structural biology. For instance, a major question is whether the overall LRRK2 structure is affected by disease-causing mutations. At the biochemical level, several studies have reported that LRRK2 disease mutations, particularly mutations in the ROC and COR domains, can affect dimerization^16, 17, 40^. Work in this area has also shown that disease mutations can affect interactions between LRRK2 domains without affecting overall dimerization, suggesting that the overall dimer structure is likely to be affected^16, 40^. Another issue to address at the structural level is the intramolecular regulation of LRRK2 functions. For instance, LRRK2 kinase activity requires the LRRK2 ROC domain but is not dependent on ROC’s GTP binding activity per se^41^. In addition, it is as yet unclear what effect LRRK2 autophosphorylation has on LRRK2 function. While autophosphorylation is not easily detected *in vivo*, at least 1 phosphorylation site S1292 is found with increased phosphorylation levels in cellular and animal models based on the LRRK2 mutant G2019S. In addition, phosphorylation of this site is increased in urinary exosomes of both LRRK2 G2019S carrying and sporadic PD patients^42^.

In conclusion, we present a comparative cryo-EM-based insight into the 3D structures of full length LRRK2 and LRRK1. Our results reveal that LRRK2 is stabilized in solution by high pH buffers and that both LRRK2 and LRRK1 display similar, compact two-fold symmetric dimer structures with the particularity for LRRK2 that its N-terminal ARM domain extends outside of the dimer core. This work will further assist follow-up work towards high-resolution structures of these important molecules.

## Materials and Methods

### Protein expression and purification

#### Constructs

Constructs for the mammalian expression of 3xflag-LRRK2 and 3xflag-LRRK1 included the pCHMWS-3xFLAG-LRRK2 and pCHMWS-3xFLAG-LRRK1 constructs, described previously^16, 20^, as well as the p3xFLAG-CMV-10-LRRK2 construct (a generous gift from Prof. Takeshi Iwatsubo) described in ref. 43.

#### Cell culture and transfection

HEK293FT cells (Life Technologies) were cultured in 15 cm Corning petri dishes (Sigma), at 37 °C in an atmosphere containing 5% CO_2_. The culture medium was comprised of Dulbecco’s Modified Eagle’s Medium (DMEM), 10% fetal bovine serum, 0.1 mM MEM nonessential amino acids (NEAA), 2 mM L-glutamax and 1 mM MEM sodium pyruvate. One day prior to transfection, ~12.10^5^ cells were plated out on a single 15 cm petri dish. The next day, cells were transfected with 30 μg of plasmid (3xFLAG-LRRK2 or 3xFLAG-LRRK1) complexed with polyethyleneimine solution (PEI, 1 mg/ml, pH 7.0). To verify the efficacy of the transfection, a transfection with GFP plasmid was performed in parallel as a control. The proportion of cells transfected was monitored by fluorescence microscopy and the experiment was only continued for transfection rates greater than 50%.

#### Protein Purification

Protein purification was performed essentially as described previously^4, 20^. Forty-eight hours after transfection with the 3xFLAG-LRRK2 and 3xFLAG-LRRK1 constructs, the cells were rinsed with phosphate-buffered saline (PBS) and lysed in 1 mL lysis buffer (lysis buffer composition: 20 mM Tris/HCl, pH 7.5, 150 mM NaCl, 1 mM EDTA, 1% (v/v) Triton X-100, 10% (v/v) glycerol and protease inhibitor cocktail (Sigma), or the optimal composition indicated by ProteoPlex, as detailed in the *Results* section), on ice, for 10 min. The collected lysate was centrifuged at 4 °C for 10 min at 20,000 g to remove cell debris. The clear supernatant was incubated overnight at 4 °C on a rotator mixer (STARLAB, Germany) with anti-Flag M2 agarose beads (Sigma-Aldrich) that had previously been equilibrated with lysis buffer. Afterwards, the beads were washed four times at 4 °C with wash buffer (25 mM Tris/HCl pH 7.5, 400 mM NaCl, 1% (v/v) Triton X-100 or the optimal lysis/binding buffer indicated by ProteoPlex). Proteins prepared for testing in ProteoPlex assays were stored in storage buffer (*i*.*e*., 20 mM Tris/HCl pH 7.4, 200 mM NaCl, 5 mM MgCl_2_ 1 mM DTT, 0.02% (v/v) Triton X-100, 50% (v/v) glycerol or the optimal lysis/binding buffer determined by the preceding ProteoPlex screen supplemented with 50% (v/v) glycerol) with 3xflag-LRRK2 still bound, at 4 °C until use. For EM analysis, the affinity-bound 3xflag-LRRK2 or 3xflag-LRRK1 protein was eluted by adding 5 volumes of elution buffer containing 100 μg/ml of 3X FLAG^**®**^ peptide (Sigma) and rotating the mixture on a wheel at 4 °C for 30 minutes (STARLAB, Germany). The composition of the elution buffer was 20 mM Tris/HCl pH 7.4, 200 mM NaCl, 5 mM MgCl_2_ 1 mM DTT, 0.02% (v/v) Triton X-100, or the optimal composition indicated by ProteoPlex (see *Results* section). The beads were then spun down at 400 g for 2 minutes and supernatant containing the eluted protein was collected. EM-grids holding the purified protein were prepared straight away.

#### Radiometric nucleotide binding assay

To assess GDP/GTP binding capacity of purified 3xFLAG-LRRK1 and 3xFLAG-LRRK2, affinity resin bound protein was prepared as described under protein purification, rinsed in guanine nucleotide binding buffer (Tris 25 mM pH 7.5, NaCl 150 mM, EDTA 5 mM, Triton 0.02%) and incubated with radioactively labelled GTP (GTP-α-^33^P), in the presence or absence of 200 µM ‘cold’ nucleotides (GDP and GTP) at room temperature. Excess nucleotides were then removed by rinsing beads 3 times in nucleotide wash buffer (Tris 25 mM pH 7.5, MgCl_2_ 10 mM, dithiothreitol (DTT) 2 mM, Triton 0.02%, beta-glycerophosphate 5 mM, Na_3_VO_4_ 0.1 mM). Bound GTP-α-^33^P was measured via scintillation counting and values of the different test conditions were normalized to the binding condition without cold nucleotides.

### Buffer optimization by ProteoPlex screening

The ProteoPlex screening method based on the Thermofluor^®^ differential scanning fluorimetry assay, was used to assess the stability of the multidomain 3xflag-LRRK2 complexes in different buffer and additive systems. Conditions that resulted in thermal protein unfolding transitions closest to two state unfolding (*i*.*e*., unfolding from the native to the unfolded state, without the formation of metastable intermediates) were considered optimal, as they indicate monodispersity and increased stability of macromolecular complexes^29^. It should be noted that the ProteoPlex assays were performed with protein in the absence of Triton X-100 as it interferes with the assay signal.

ProteoPlex assays of (i) 88 buffers and (ii) 88 buffer ligands/additives were performed in 96-well plates as given in ref. 29 to determine the optimal conditions for the stability of 3xflag-LRRK2 bound to the affinity beads (*i*.*e*., the optimum lysis/binding buffer). Flag-M2 agarose beads loaded with 3xflag-LRRK2 in storage buffer as described above (see above), were rinsed at 4 °C. The rinse buffer for (i), the buffer screen, was 20 mM Tris/HCl pH 7.4, 200 mM NaCl, 5 mM MgCl_2_, 1 mM DTT. The rinse buffer for (ii), the ligand/additive screen, was the same composition as (i) with the replacement of Tris by the optimal buffer determined from the buffer screen (Hepes pH 6.8, see *Results* section) at 4 °C, The rinsed beads were immediately aliquoted (16 µl aliquots) and tested by addition of 2 µl of ProteoPlex ×88 buffers (or ×88 ligands) screens at 4 °C. Then 2 µl SYPRO^**®**^ Orange (Sigma Aldrich) was added to each aliquot and melting curves (*i*.*e*., state transition graphs) were recorded for each condition to assess the stability of the bound protein.

To determine optimal buffer for eluting LRRK2 from affinity beads, flag-M2 agarose beads binding LRRK2 were washed 3 times with 3 beads volumes of the optimal binding buffer (HEPES pH 6.8, 5 mM MgCl_2_, 100 mM NH_4_Cl, 5% (v/v) Ethylen Glycol and 10 mM CaCl_2_) and spun down over 1 minute at 4 °C and the supernatant discarded resulting in the same initial beads volume (600 µl). Aliquots (24×) were deposited in a transparent well plate and rinsed in the ProteoPlex assay buffers (sodium citrate, HEPES or Tris buffers with pHs ranging from 5.5 to 9) finally leaving a 25 µl aliquot at 4 °C. 3xflag-LRRK2 protein was eluted at 4 °C by the addition of 1 µl 3X FLAG^**®**^ peptide to each well (to 100 µg/ml). After 30 minutes the beads were sedimented using a table top centrifuge for 1 minute at 2500 rpm at 4 °C, and 18 µl of supernatant containing eluted 3xflag-LRRK2 protein were collected. 2 µl of SYPRO^**®**^ Orange dye was added to each supernatant following the ProteoPlex protocol and melting curves were recorded for each buffer condition. The curves were visually assessed, as the signals were too weak to carry out the standard ProteoPlex automated analysis described in ref. 29. Again, the buffer conditions displaying thermal unfolding transitions closest to a two state unfolding, that are known to be indicative of monodispersity and stability of macromolecular complexes^29^, were used to adapt the LRRK2 elution buffer, as indicated in the *Results* section.

### Electron microscopy

For negative stain transmission electron microscopy (TEM), 3.5 µl of purified protein sample was pipetted onto a glow-discharged, carbon-coated copper grid and left to adsorb for 1 minute. The grid was then washed on three droplets of milliQ water, and subsequently stained on two droplets of 2% uranyl acetate (1 minute per drop), blotting between each step. Grids were scanned using a Philips CM10 TEM (FEI Company, Eindhoven, The Netherlands) operated at 80 kV under low-dose conditions (20 electrons/Å^2^). Images were collected at a nominal magnification of 96,000x with defocus values varying between −0.5 to −1.5 µm and recorded by a 2 K Veleta side-mounted TEM CCD camera (Olympus), corresponding to a pixel size of 3.8 Å at the specimen level.

For cryo-EM, 3.5 µl of purified protein sample was pipetted onto a glow-discharged, holey carbon film (Quantifoil R2/2, Quantifoil Micro Tools, Jena, Germany) with an additional thin layer of carbon, and left to adsorb for 1 minute. The grids were then rapidly plunge-frozen in liquid ethane cooled by liquid nitrogen, using a MarkIV Vitrobot (FEI, Eindhoven, Netherlands). The time between elution of purified LRRK2 or LRRK1 and cryo-EM grid preparation was kept to a minimum (60 minutes). The frozen grids were transferred to a Gatan-626 cryo-holder. Micrographs were recorded under low-dose conditions (25 electrons/Å^2^) using a CM200 TEM (FEI Company, Eindhoven, The Netherlands) equipped with a TVIPS F416 CMOS camera (TVIPS, Gauting, Germany). The microscope was operated at 200 kV and a nominal magnification of 58,000x. The defocus ranged from −1 to −2.5 µm, corresponding to a pixel size of 2.7 Å at the specimen level.

### Image processing

The EMAN2 software suite^43^ was initially used. Particles, *i*.*e*., images of single LRRK2 or LRRK1 complexes, were extracted from the negative stain TEM or cryo-EM micrographs using the e2boxer interactive procedure. Contrast transfer function (CTF) correction was accomplished by producing high-pass filtered and phase-flipped particles, using the e2ctf program. Reference-free class averages were generated using the e2refine2d program, requesting the generation of 128 classes with a maximum of 100 particles per class. The e2initialmodel utility was employed to generate initial models from the best 2D class averages (10 in all), without and with imposed C2 symmetry. The final 3D maps were refined using the standard iterative projection matching, class-averaging and Fourier reconstruction procedure of EMAN2. The resolution of each reconstruction was determined by the gold standard Fourier shell correlation criterion. UCSF Chimera^44^ was employed to visualize and analyze the final maps.

In addition, the RELION software^33^ was employed to generate 2D class averages of the same particles and obtain a 3D model for comparison. After CTF correction in EMAN2, the newly incorporated e2refinetorelion2d EMAN2 functionality^45^ was used to export the particles to RELION and run 25 rounds of 2D reference-free class refinement with 128 classes in total, finishing with an angular sampling of 1.875 degrees translational sampling of 0.5 pixels. To generate an initial model in RELION, a 3D classification was performed with only one class and C1 symmetry, using a sphere as the initial reference and all the selected particles from the 2D classification. After 50 iterations, an initial consensus model with a spectral signal-to-noise ratio (SSNR) below 1.0 at 19.2 Å was obtained. Using this consensus model as reference, 3D classification was performed using four classes with C2 symmetry. Initially, 50 iterations were performed with a Tau factor of 3.6 and a sampling of, respectively, 7.5 degrees and 1 pixel. Afterwards, an additional 25 steps were performed using an angular step of 1.9 degrees.

## Electronic supplementary material

Supplemental information and figures
