# Supplementary material for: Cryo-EM analysis of homodimeric full-length LRRK2 and LRRK1 protein complexes
Source: Sci Rep. 2017 Aug 17;7:8667. doi: 10.1038/s41598-017-09126-z (PMC5561129; doi:10.1038/s41598-017-09126-z)
Supplement: Supplementary file 1 — Supplemental information and figures [file 41598_2017_9126_MOESM1_ESM.pdf]

Supplemental Material

for

Cryo-EM analysis of homodimeric full-length LRRK2 and LRRK1 protein complexes

Kushal Sejwal[1], Mohamed Chami[1], Hervé Rémigy[2], Renée Vancraenenbroeck[3,§], William Sibran[4], Rosmarie Sütterlin[1], Paul Baumgartner[1], Robert McLeod[1], Marie-Christine Chartier-Harlin[4], Veerle Baekelandt[3], Henning Stahlberg[1,*] and Jean-Marc Taymans[3,4*]

[1] *Center for Cellular Imaging and NanoAnalytics (C-CINA), Biozentrum, University of Basel, Basel 4056, Switzerland*

[2] *FEI Company, Eindhoven, The Netherlands.*

[3] *KU Leuven, Laboratory for Neurobiology and Gene Therapy, Department of Neurosciences, 3000 Leuven, Belgium*

[4] *Université de Lille, Inserm, CHU Lille, UMR-S1172 - JPArc - Centre de Recherche Jean-Pierre AUBERT Neurosciences et Cancer, F-59000 Lille, France*

§ *Current address: Department of Structural Biology, Weizmann Institute of Science, Rehovot 76100, Israel*

* *Please send correspondence to Henning Stahlberg, C-CINA, Biozentrum, University Basel, Mattenstrasse 26, CH-4058 Basel, Switzerland, Tel. +41-61-387 32 62, henning.stahlberg@unibas.ch; and Jean-Marc Taymans, Jean-Pierre Aubert Research Center, UMR-S1172, Early Stages of Parkinson's disease, Place de Verdun, 59045 Lille, France, Tel. +33 3 20 29 88 68, jean-marc.taymans@inserm.fr.*

Sequence alignment of the LRRK2 N-terminal region (amino acids 1 to 979) with the LRRK1 N-terminal region (amino acids 1 to 275)

Secondary structure elements (NetSurfP [1]) are indicated: α-helices are red *italic*, and β-strands yellow underlined. Putative phosphorylation sites[#1] described by at least two different groups are double underlined.

In this region, identity rate between LRRK1 and LRRK2 is 4 % (44 / 983, marked with an asterisk (*)), similarity rate is 9 % (93 / 983, marked with a plus sign (+)). For the ankyrin repeats alone, sequence identity and similarity are 14 % (44 / 313) and 30 % (93 / 313), respectively.

```
                     10        20        30        40        50        60
HsLRRK2_N   MASGSCQGCEEDEETLKKLIVRLNNVQEGKQIETLVQILEDLLVFTYSEHASKLFQGKNI
HsLRRK1_N   ------------------------------------------------------------

                     70        80        90       100       110       120
HsLRRK2_N   HVPLLIVLDSYMRVASVQQVGWSLLCKLIEVCPGTMQSLMGPQDVGNDWEVLGVHQLILK
HsLRRK1_N   ------------------------------------------------------------

                    130       140       150       160       170       180
HsLRRK2_N   MLTVHNASVNLSVIGLKTLDLLLTSGKITLLILDEESDIFMLIFDAMHSFPANDEVQKLG
HsLRRK1_N   ------------------------------------------------------------

                    190       200       210       220       230       240
HsLRRK2_N   CKALHVLFERVSEEQLTEFVENKDYMILLSALTNFKDEEEIVLHVLHCLHSLAIPCNNVE
HsLRRK1_N   ------------------------------------------------------------

                    250       260       270       280       290       300
HsLRRK2_N   VLMSGNVRCYNIVVEAMKAFPMSERIQEVSCCLLHRLTLGNFFNILVLNEVHEFVVKAVQ
HsLRRK1_N   ------------------------------------------------------------

                    310       320       330       340       350       360
HsLRRK2_N   QYPENAALQISALSCLALLTETIFLNQDLEEKNENQENDDEGEEDKLFWLEACYKALTWH
HsLRRK1_N   ------------------------------------------------------------

                    370       380       390       400       410       420
HsLRRK2_N   RKNKHVQEAACWALNNLLMYQNSLHEKIGDEDGHFPAHREVMLSMLMHSSSKEVFQASAN
HsLRRK1_N   ------------------------------------------------------------
```

[#1]) S4 [2], S5 [2], T424 [2], T524 [2], T776 [2], T826 [2], T833 [2], T838 [2], S850 [2], S858 [2], S860 [2,3], S865 [2], S895 [2], S898 ([2], S908 [2], S910 [2-5], S912 [2,4], S926 [2], S933 [2], S935 [2-4], S954 [2], S955 [2,3,5], S958 [2], S971 [2], S973 [2-5], S975 [2], S976 [2,3], S979 [2]

```
                  430       440       450       460       470       480
HsLRRK2_N  ALSTLLEQNVNFRKILLSKGIHLNVLELMQKHIHSPEVAESGCKMLNHLFEGSNTSLDIM
HsLRRK1_N  ------------------------------------------------------------

                  490       500       510       520       530       540
HsLRRK2_N  AAVVPKILTVMKRHETSLPVQLEALRAILHFIVPGMPEESREDTEFHHKLNMVKKQCFKN
HsLRRK1_N  ------------------------------------------------------------

                  550       560       570       580       590       600
HsLRRK2_N  DIHKLVLAALNRFIGNPGIQKCGLKVISSIVHFPDALEMLSLEGAMDSVLHTLQMYPDDQ
HsLRRK1_N  ------------------------------------------------------------

                  610       620       630       640       650       660
HsLRRK2_N  EIQCLGLSLIGYLITKKNVFIGTGHLLAKILVSSLYRFKDVAEIQTKGFQTILAILKLSA
HsLRRK1_N  ------------------------------------------------------------

                  670       680       690       700       710       720
HsLRRK2_N  SFSKLLVHHSFDLVIFHQMSSNIMEQKDQQFLNLC-CKCFAKVAMDDYLKNVMLERACDQ
HsLRRK1_N  -----------------------MAGMSQRPPSMYWCVGPEESAVCPERAMETLNGAGDT
                                  *    *+  ++  *    + *+        *  * *

                  730       740       750       760       770       780
HsLRRK2_N  NNSIMVECLLLLGADANQAKEGSSLICQVCEKESSPKLVELLLNSGSREQDVRKALTISI
HsLRRK1_N  GGKPSTRG--GDPAARSRRTEGIRAAYRRGDRGGARDLLEEACDQCASQLEKGQLLSIPA
                        *  ++  **     +  ++  +  *+*   +  + + +  + *+*

                  790       800       810       820       830       840
HsLRRK2_N  GKGDSQIISLLLRRLALDVANNSICLGGFCIGKV--EPSWLGPLFPDKTSNLRKQTNIAS
HsLRRK1_N  AYGDLEMVRYLLSKRLVELPTEPTDDNPAVVAAYFGHTAVVQELLESLPGPCSPQRLLNW
             ** +++  ** +  +++           +       + +  *          *  +

                  850       860       870       880       890       900
HsLRRK2_N  TLARMVIRYQMKSAVEEGTASGSDGNFSEDVLSKFDEWTFIPDSSMDSVFAQSDDLDSEG
HsLRRK1_N  MLALACQRGHLGVVKLLVLTHGAD---PESYAVRKNEFPVIVRLPLYAAIKSGNE-----
            **    *  +          *+*    *    + +*+  *    + +     ++

                  910       920       930       940       950       960
HsLRRK2_N  SEGSFLVKKKSNSISVGEFYRDAVLQRCSPNLQRHSNSLGPIFDHEDLLKRKRKILSSDD
HsLRRK1_N  DIAIFLLRH-------GAYFCSYILLD-SPDPSKH--LLRKYFIEASPLPSSYPGKT---
               **++        * ++   +*   **+  +*   *   *     *       +

                  970       980
HsLRRK2_N  SLRSSKLQSHMRHS-DSISSLAS
HsLRRK1_N  ALRVK--WSHLRLPWVDLDWLID
           +**     **+*     +  *
```

Sequence alignment of the LRRK2 armadillo repeats

In the N-terminal region of LRRK2 (amino acids 1 to 659), we could detect the presence of 14 putative armadillo repeats. The consensus sequence was calculated such that residues occurring in (more than) 50 % of the repeats are given. Residue conservation is coloured as explained in the consensus (C) sequence (X = any residue, ϕ = hydrophobic residue (A, F, I, L, M, V, W; blue), + = charged residue (D, E, H, K, R; violet), $ = polar, non-charged residue (N, Q, S, T, Y; orange), £ = special amino acids (P, C, G; brown)) and - is a possible insertion or deletion site. Secondary structure elements (NetSurfP [1]) are indicated: α-helices are red *italic*, no β-strands were found.

LRRK2 ARMADILLO REPEATS

**1**	MA*SG*	*S*	*C*	*Q*	*G*	*C*	*E*	*E*	*D*	*E*	-	*E*	*T*	*L*	*K*	*K*	*L*	*I*	*V*	*R*	*L*	*N*	*N*	V	Q	E	*G*	*K*	*Q*	*I*	*E*	*T*	*L*	-	*V*	*Q*	*I*	*L*	*E*	*D*	*L*	*V*	*V*	*F*	T	-
**2**	YS*E*	*H*	*A*	*S*	*K*	*L*	*F*	*Q*	*G*	K	-	N	I	*H*	*V*	*P*	*L*	*L*	*I*	*V*	*L*	*D*	S	Y	M	R	*V*	*A*	*S*	*V*	*Q*	*Q*	*V*	*G*	*W*	*S*	*L*	*L*	*C*	*K*	*L*	*I*	*E*	*V*	C	-
**3**	*PG*	*T*	*M*	*Q*	*S*	*L*	*M*	G	P	Q	DVGNDWE*VL*	*G*	*V*	*H*	*Q*	*L*	*I*	*L*	*K*	*M*	*L*	*T*	*V*	H	N	A	S	*V*	*N*	*L*	*S*	*V*	*I*	*G*	*L*	*K*	*T*	*L*	*D*	*L*	*L*	*L*	*T*	S	-	-
**4**	G	K	I	T	L	L	*I*	*L*	*D*	E	ES	D	*I*	*F*	*M*	*L*	*I*	*F*	*D*	*A*	*M*	*H*	S	F	P	A	N	*D*	*E*	*V*	*Q*	*K*	*L*	*G*	*C*	*K*	*A*	*L*	*H*	*V*	*L*	*F*	*E*	R	V	-
**5**	SE*E*	*Q*	*L*	*T*	*E*	*F*	*V*	*E*	N	K	-	-	D	*Y*	*M*	*I*	*L*	*L*	*S*	*A*	*L*	*T*	N	F	K	D	E	*E*	*E*	*I*	*V*	*L*	*H*	*V*	*L*	*H*	*C*	*L*	*H*	*S*	*L*	*A*	I	P	C	-
**6**	N	*N*	*V*	*E*	*V*	*L*	*M*	*S*	G	N	V	R	*C*	*Y*	*N*	*I*	*V*	*V*	*E*	*A*	*M*	*K*	*A*	F	P	M	S	*E*	*R*	*I*	*Q*	*E*	*V*	*S*	*C*	*C*	*L*	*L*	*H*	*R*	*L*	*T*	L	G	-	-
**7**	-	N	F	F	N	*I*	*L*	*V*	*L*	*N*	-	*E*	*V*	*H*	*E*	*F*	*V*	*V*	*K*	*A*	*V*	*Q*	*Q*	Y	P	E	N	*A*	*A*	*L*	*Q*	*I*	*S*	*A*	*L*	*S*	*C*	*L*	*A*	*L*	*L*	*T*	*E*	*T*	*I*	**a**
**8**	E	N	D	D	E	*G*	*E*	*E*	*D*	*K*	*L*	F	*W*	*L*	*E*	*A*	*C*	*Y*	*K*	*A*	*L*	*T*	*W*	H	R	K	N	*K*	*H*	*V*	*Q*	*E*	*A*	*A*	*C*	*W*	*A*	*L*	*N*	*N*	*L*	*L*	M	Y	Q	-
**9**	N	*S*	*L*	*H*	*E*	*K*	*I*	*G*	*D*	E	DGHF	P	A	*H*	*R*	*E*	*V*	*M*	*L*	*S*	*M*	*L*	*M*	H	S	S	S	*K*	*E*	*V*	*F*	*Q*	*A*	*S*	*A*	*N*	*A*	*L*	*S*	*T*	*L*	*L*	*E*	Q	N	-
**10**	*V*	*N*	*F*	*R*	*K*	*I*	*L*	*L*	*S*	K	-	G	*I*	*H*	*L*	*N*	*V*	*L*	*E*	*L*	*M*	*Q*	*K*	I	H	H	S	*P*	*E*	*V*	*A*	*E*	*S*	*G*	*C*	*K*	*M*	*L*	*N*	*H*	*L*	*F*	E	G	S	-
**11**	N	*T*	*S*	*L*	*D*	*I*	*M*	*A*	*A*	-	-	-	*V*	*V*	*P*	*K*	*I*	*L*	*T*	*V*	*M*	*K*	*R*	H	E	T	S	*L*	*P*	*V*	*Q*	*L*	*E*	*A*	*L*	*R*	*A*	*I*	*L*	*H*	*F*	I	V	P	G	**b**
**12**	*LN*	*M*	*V*	*K*	*K*	*Q*	*C*	*F*	*K*	N	D	*I*	*I*	*H*	*K*	*L*	*V*	*L*	*A*	*A*	*L*	*N*	*R*	F	I	G	N	*P*	*G*	*I*	*Q*	*K*	*C*	*G*	*L*	*K*	*V*	*I*	*S*	*S*	*I*	V	H	F	P	-
**13**	-	D	*A*	*L*	*E*	*M*	*L*	*S*	*L*	E	-	G	A	*M*	*D*	*S*	*V*	*L*	*H*	*T*	*L*	*Q*	*M*	Y	P	D	*D*	*Q*	*E*	*I*	*Q*	*C*	*L*	*G*	*L*	*S*	*L*	*I*	*G*	*Y*	*L*	*I*	*T*	K	K	-
**14**	-	-	-	N	V	F	*I*	*G*	*T*	*G*	*H*	*L*	*L*	*A*	*K*	*I*	*L*	*V*	*S*	*S*	*L*	*Y*	R	F	K	D	V	*A*	*E*	*I*	*Q*	*T*	*K*	*G*	*F*	*Q*	*T*	*I*	*L*	*A*	*I*	*L*	K	L	S	-
**C**	-	**$**	**ϕ**	**X**	**+**	**ϕ**	**ϕ**	**X**	**X**	**+**	-	**X**	**ϕ**	**X**	**+**	**ϕ**	**ϕ**	**L**	**+**	**ϕ**	**L**	**$**	**X**	**ϕ**	**X**	**+**	**$**	**X**	**+**	**ϕ**	**Q**	**X**	**ϕ**	**G**	**ϕ**	**X**	**ϕ**	**L**	**X**	**X**	**L**	**ϕ**	**+**	**X**	**X**	-

a: *FLN*Q*DLEEKNE*NQ; **b:** MP*EESREDT*EFHHK

Sequence alignment of the LRRK2 ankyrin repeats and the LRRK1 ankyrin repeats

In the N-terminal region of LRRK2 (amino acids 672 to 861), we could detect the presence of 6 putative ankyrin repeats. In the N-terminal domain of LRRK1 (amino acids 1 to 247), we could detect the presence of 7 putative ankyrin repeats. The consensus sequences were calculated such that residues occurring in (more than) 50 % of the repeats are given. Residue conservation is coloured as explained in the consensus (C) sequences (X = any residue, ϕ = hydrophobic residue (A, F, I, L, M, V, W; blue), + = charged residue (D, E, H, K, R; violet), $ = polar, non-charged residue (N, Q, S, T, Y; orange), £ = special amino acids (P, C, G; brown)) and - is a possible insertion or deletion site. Secondary structure elements (NetSurfP [1]) are indicated: α-helices are red *italic*, no β-strands were found.

LRRK2 ANKYRIN REPEATS

1					D	L	V	I	F	H	Q	M	S	S	N	I	M	E	Q	K	D	Q	Q	F	L	N	L	C	C	K	C	F	A	K
2		V	A	M	D	D	Y	L	K	N	*V*	*M*	*L*	*E*	*R*	*A*	*C*	*D*	*Q*	*N*	*N*	*S*	*I*	*M*	*V*	*E*	*C*	*L*	*L*	*L*	*L*	G	A	D
3		A	N	Q	A	K	E	G	S	S	L	*I*	*C*	*Q*	*V*	*C*	*E*	K	E	S	S	*P*	*K*	*L*	*V*	*E*	*L*	*L*	*L*	*N*	S	G	S	R
4					E	Q	D	V	R	K	*A*	*L*	*T*	*-*	*I*	*S*	*I*	*G*	K	G	D	*S*	*Q*	*I*	*I*	*S*	*L*	*L*	*L*	*R*	*R*	L	A	L
5				D	V	A	N	N	S	I	C	L	G	G	F	C	I	G	K	V	E	P	S	W	*L*	*G*	*P*	*L*	F	P	D	K	T	S
6	N	L	R	K	Q	T	N	I	A	S	T	*L*	*A*	*R*	*M*	*V*	*I*	*R*	*Y*	*Q*	M	K	S	A	V	E	E	G	T	A	S	G	S	D
C	-	ϕ	X	X	+	X	$	ϕ	$	$	ϕ	L	ϕ	X	ϕ	ϕ	I	+	+/$	$	+	$	$	ϕ	V	E	L	L	L	X	X	G	A	+

LRRK1 ANKYRIN REPEATS

1	RPPSMYWC	V	G	P	E	E	S	A	V	C	*P*	*E*	*R*	*A*	*M*	*E*	*T*	*L*	N	G	A	*G*	*D*	*T*	*G*	*G*	*K*	*P*	*S*	*T*	R	G	G	D
2		P	A	A	R	S	R	R	T	E	*G*	*I*	*-*	*R*	*A*	*A*	*Y*	*R*	R	G	D	*R*	*G*	*G*	*A*	*R*	*D*	*L*	*L*	*E*	E	A	C	D
3		Q	C	A	S	Q	L	E	K	G	Q	*L*	*L*	*S*	*I*	*P*	*A*	*A*	Y	G	D	*L*	*E*	*M*	*V*	*R*	*Y*	*L*	*L*	*S*	*K*	R	L	V
4		E	L	P	T	E	P	T	D	D	N	*P*	*A*	*V*	*V*	*A*	*A*	*Y*	F	G	H	*T*	*A*	*V*	*V*	*Q*	*E*	*L*	*L*	*E*	*S*	L	P	G
5		P	C	S	P	Q	R	L	L	N	W	*M*	*L*	*A*	*L*	*A*	*C*	*Q*	R	G	H	*L*	*G*	*V*	*V*	*K*	*L*	*L*	*V*	*L*	*T*	H	G	A
6	DPESYAVR	K	N	E	F	P	V	I	V	R	L	*P*	*L*	*Y*	*A*	*A*	*I*	*K*	S	G	N	*E*	*D*	*I*	*A*	*I*	*F*	*L*	*L*	*R*	H	G	A	Y
7		-	-	-	-	-	F	C	S	Y	I	*L*	*L*	*D*	*S*	*P*	*D*	*P*	S	K	H	*L*	*L*	*R*	*K*	*Y*	*F*	*I*	*E*	A	S	P	L	P
C	-	X	X	X	X	X	X	X	X	X	X	ϕ	L	X	ϕ	A	X	X	$	G	+	X	X	ϕ	ϕ	X	X	L	L	X	+	X	£	X

Sequence alignment of the LRRK2 LRR domain (amino acids 980 to 1319) with the LRRK1 LRR domain (amino acids 276 to 623)

Secondary structure elements (NetSurfP [1]) are indicated: α-helices are red *italic*, and β-strands yellow underlined. Putative phosphorylation sites[#1] described by at least two different groups are double underlined. The LRR domains share 23 % (85 / 357) sequence identity and 41 % (147 / 357) sequence similarity. Identical residues are marked with an asterisk (*) and similar residues are marked with a plus sign (+).

```
                     10        20        30        40        50        60
HsLRRK2_LRR   EREYITSLDLSANELRDIDALSQKCCISVHLEHLEKLELHQNALTSFP--QQLCETLKS-
HsLRRK1_LRR   ISCQITELDLSANCLATLPS-----VIPWGLINLRKLNLSDNHLGELPGVQSSDEIICSR
                  ** ****** *  + +      *   * +* ** *  * *   *  *   * + *

                     70        80        90       100       110       120
HsLRRK2_LRR   LTHLDLHSNKFTSFPSYLLKMSCIANLDVSRNDIGPSVV----LDPTVKCPTLKQFNLSY
HsLRRK1_LRR   LLEIDISSNKLSHLPPGFLHLSKLQKLTASKNCL-EKLFEEENATNWIGLRKLQELDISD
              *  +*+ *** +  *   * +* +  *  *+* +   +         +    *++ ++*

                    130       140       150       160       170       180
HsLRRK2_LRR   NQLSFVPENLTDVVEKLEQLILEGNKISGICSPLRLKELKILNLSKNHISSLSENFLEAC
HsLRRK1_LRR   NKLTELPALFLHSFKSLNSLNVSRNNLKVFPDPW-ACPLKCCKASRNALECLPDKMAVFW
              *+*+ +*       + *  * +  * +     *     **    *+* +  * +

                    190       200       210       220       230       240
HsLRRK2_LRR   P-KVESFSARMNFLAAMP---FLPPSMTILKLSQNKFSCIP-EAILNLPHLRSLDMSSND
HsLRRK1_LRR   KNHLKDVDFSENALKEVPLGLFQLDALMFLRLQGNQLAALPPQEKWTCRQLKTLDLSRNQ
                 ++      * *  +*   *  ++   *+*  *+ + +* +       *++**+* *

                    250       260       270       280       290       300
HsLRRK2_LRR   IQYLPGPAHWKSLNLRELL-FSHNQI---SILDLSEKAYLWSRVEKLHLSHNKLKEIPPE
HsLRRK1_LRR   LGKNEDGL--KTKRIAFFTTRGRQRSGTEAASVLEFPAFLSESLEVLCLNDNHLDTVPPS
              +         *+  +         +    +   *   *+*   +* * *+ * *  +**

                    310       320       330       340       350
HsLRRK2_LRR   IGCLENLTSLDVSYNLELRSFPNEMGKLSKIWDLPLDELHLNFDFKHIG-CKAKDII
HsLRRK1_LRR   VCLLKSLSELYLGNNPGLRELPPELGQLGNLWQLDTEDLTISNVPAEIQKEGPKAML
              +  *++*+ * +  *  **  * *+*+*  +* *  ++* ++     *     * ++
```

[#1]) T1024 [6], S1025 [6], S1058 [5], S1124 [2], S1253 [7], S1283 [7], S1292 [2,7]

Sequence alignment of the LRRK2 LRRs and the LRRK1 LRRs

We could detect the presence of 14 putative LRRs for LRRK2 (amino acids 980 to 1319) and LRRK1 (amino acids 275 to 623). The consensus sequence was calculated such that residues occurring in (more than) 50 % of the repeats are given. Residue conservation is coloured as explained in the consensus (C) sequence (X = any residue, ϕ = hydrophobic residue (A, F, I, L, M, V, W; blue), + = charged residue (D, E, H, K, R; violet), $ = polar, non-charged residue (N, Q, S, T, Y; orange), £ = special amino acids (P, C, G; brown)), - is a possible insertion or deletion site and var is a variable region.

LRRK2 LRR REPEATS

1	Y	I	T	S	L	D	L	S	A	N	-	E	L	-	R	D	I	D	ALSQKCCISVHLE
2	H	L	E	K	L	E	L	H	Q	N	-	A	L	-	T	S	F	P	QQLCETLK
3	S	L	T	H	L	D	L	H	S	N	-	K	F	-	T	S	F	P	SYLLKMS
4	C	I	A	N	L	D	V	S	R	N	-	D	I	G	P	S	V	V	LDPTVKCP
5	T	L	K	Q	F	N	L	S	Y	N	-	Q	L	-	S	F	V	P	ENLTDVVE
6	K	L	E	Q	L	I	L	E	G	N	-	K	I	-	S	G	I	C	SPLRLK
7	E	L	K	I	L	N	L	S	K	N	-	H	I	-	S	S	L	S	ENFLEACP
8	K	V	E	S	F	S	A	R	M	N	-	F	L	-	A	A	M	P	FLPP
9	S	M	T	I	L	K	L	S	Q	N	-	K	F	-	S	C	I	P	EAILNLP
10	H	L	R	S	L	D	M	S	S	N	-	D	I	-	Q	Y	L	P	GPAHWKSL
11	N	L	R	E	L	L	F	S	H	N	-	Q	I	-	S	I	L	D	LSEKAYLWS
12	R	V	E	K	L	H	L	S	H	N	-	K	L	-	K	E	I	P	PEIGCLE
13	N	L	T	S	L	D	V	S	Y	N	L	E	L	-	R	S	F	P	NEMGKLS
14	K	I	W	D	L	P	L	D	E	L	-	H	L	N	F	D	F	K	HIGCKAKDII
**C**	**+**	**L**	**+**	**$**	**L**	**+**	**L**	**S**	**X**	**N**	-	**+**	**L**	-	**$**	**X**	**ϕ**	**P**	**var**

LRRK1 LRR REPEATS

1	Q	I	T	E	L	D	-	L	S	A	N	-	C	L	-	A	T	L	P	SVIPWGLI
2	N	L	R	K	L	N	-	L	S	D	N	-	H	L	-	G	E	L	P	GVQSSDEIICS
3	R	L	L	E	I	D	-	I	S	S	N	-	K	L	-	S	H	L	P	PGFLHLS
4	K	L	Q	K	L	T	-	A	S	K	N	-	C	L	-	E	K	L	F	EEENATNWIGLR
5	K	L	Q	E	L	D	-	I	S	D	N	-	K	L	-	T	E	L	P	ALFLHSFK
6	S	L	N	S	L	N	-	V	S	R	N	-	N	L	-	K	V	F	P	DPWAC
7	P	L	K	C	C	K	-	A	S	R	N	-	A	L	-	E	C	L	P	DKMAVFWKN
8	H	L	K	D	V	D	-	F	S	E	N	-	A	L	-	K	E	V	P	LGLFQLD
9	A	L	M	F	L	R	-	L	Q	G	N	-	Q	L	-	A	A	L	P	PQEKWTCR
10	Q	L	K	T	L	D	-	L	S	R	N	-	Q	L	-	G	K	N	E	DGLKTK
11	R	I	A	F	F	T	T	R	G	R	Q	-	R	S	GTEAASV	L	E	F	P	AFLSE
12	S	L	E	V	L	C	-	L	N	D	N	-	H	L	-	D	V	T	P	PSVCLLK
13	S	L	S	E	L	Y	-	L	G	N	N	P	G	L	-	R	E	L	P	PELGQLG
14	N	L	W	Q	L	D	-	T	E	D	L	-	T	I	-	S	N	V	P	AEIQKEGPKAML
**C**	**$**	**L**	**X**	**+**	**L**	**+**	-	**ϕ**	**S**	**+**	**N**	-	**X**	**L**	-	**X**	**+**	**L**	**P**	**var**

Sequence alignment of the LRRK2 ROC-COR bi-domain (amino acids 1320 to 1844) with the LRRK1 ROC-COR bi-domain (amino acids 624 to 1207)

Secondary structure elements (NetSurfP [1]) are indicated: α-helices are red *italic*, and β-strands yellow underlined. LRRK2 pathogenic mutations (R1441C/G and Y1699C), LRRK2 risk factor (R1628P) and LRRK2 functional mutations (K1347A (G1 box motif)) and T1348N (G1 box motif)) are boxed. Position 1516 (coloured blue), indicates the approximate position transition area between ROC and COR. Putative phosphorylation sites[#1] described by at least two different groups are double underlined. The G box elements are highlighted in **bold** and numbered (G1 to G5).

For the ROC-COR bi-domain, identity rate between LRRK1 and LRRK2 is 26 % (154 / 586; marked with an asterisk (*)), similarity rate is 46 % (270 / 586; marked with a plus sign (+)). The identity and similarity scores for the ROC domain are 27 % and 48 % respectively. For the COR domain, we calculated 25 % identity and 44 % similarity.

ROC GTPases have slightly different GDP / GTP-binding motifs (G box elements) than other small GTPases. For example, the G4 box motif is not [TN][KQ]XD[2] but [TN][KH]XD and their G5 box motif is not [TGC][CS]A[KLT] but XX[CSA]X [8]. In addition, they have an extra N-terminal helix not seen in other small GTPases [8,9]. This helix is predicted to span residues 1312 to 1326 in LRRK2 [9], which makes it, according to our analysis, part of the LRR and the ROC domain.

[#1]) Y1332 [7], T1343 [2,6,7,10], S1345 [2,6,7], T1348 [2,6,10], T1349 [6], T1357 [7], T1368 [2,7,10], Y1402 [7], S1403 [6,7,11], T1404 [6,7,11], T1410 [2,6,7,10,11], S1443 [7], S1444 [7], S1445 [7], T1452 [2,6,7], S1457 [7], S1467 [7], T1470 [7], Y1485 [7], T1491 [2,6,7,11], T1503 [2,6,7,10], S1508 [7], S1536 [7], T1612 [7], S1647 [7]

[2]) Square brackets, [], mean any one of the enclosed amino acid residues.

```
                    10         20         30         40         50         60
                                       G1 box                   G2 box
HsLRRK2_RC  RFLQQRLKKAVPYNRMKLMIVGNTGSGKTTLLQQLMKTKKSDLGMQSATVGIDVKDWPIQ
HsLRRK1_RC  SYLRAQLRKAEKCKLMKMIIVGPPRQGKSTLLEILQTGRAPQVVHGEATIRTTKWELQRP
             +*+ +*+**     **+****     **+****+ *    +    +     **+      +

                    70         80         90        100        110        120
                                 G3 box
HsLRRK2_RC  IRDKRKRD-LVLNVWDFAGREEFYSTHPHFMTQRALYLAVYDLSKGQAEVDAMKPWLFNI
HsLRRK1_RC  AGSRAKVESVEFNVWDIGGPASMATVNQCFFTDKALYVVVWNLALGEEAVANLQFWLLNI
               + * + +  ****  *       + +   *  *  +****+ *++*+ *+   *   ++ ** **

                   130        140        150        160        170        180
                               G4 box
HsLRRK2_RC  KARASSSPVILVGTHLDVSDEKQRKACMSKITKELLNKR---------GFPAIRDYHFVN
HsLRRK1_RC  EAKAPNAVVLVVGTHLDLIEAKFRVERIATLRAYVLALCRSPSGSRATGFPDITFKHLHE
            +*+* ++ *++*******+ + * *    ++ +   +*             *** *   *

                   190        200        210        220        230        240
            G5 box
HsLRRK2_RC  ATEES-DALAKLRKTIINESLNFK-----IRDQLVVGQLIPDCYVELEKIILSE--RKNV
HsLRRK1_RC  ISCKSLEGQEGLRQLIFHVTCSMKDVGSTIGCQRLAGRLIPRSYLSLQEAVLAEQQRRSR
             + +* +    **+ * + + + *       *  * + *+***  *+ *++ +*+*  *++

                   250        260        270        280        290        300
HsLRRK2_RC  PIEFPVIDRKRLLQLVREN--QLQLDENELPHAVHFLNESGVLLHFQDPALQLSDLYFVE
HsLRRK1_RC  DDDVQYLTDRQLEQLVEQTPDNDIKDYEDLQSAISFLIETGTLLHFPDTSHGLRNLYFLD
              +    +  ++* *** +         *  +*   *+ ** *+* **** * +   * +****++

                   310        320        330        340        350        360
HsLRRK2_RC  PKWLCKIMAQILTVKVEGCPKHPKGIISRRDVEKFLSKKRKFPKNYMSQYFKLLEKFQIA
HsLRRK1_RC  PIWLSECLQRIFNIKGS-RSVAKNGVIRAEDLRMLLVGTG-FTQQTEEQYFQFLAKFEIA
            * ** + + +*  +*          *+*   *+    *        *  +    ***+ * **+**

                   370        380        390        400        410        420
HsLRRK2_RC  LPIGEEYLLVPSSLSDHRPVIELPHCE-NSEIIIRLYEMPYFPMGFWSRLINRLL-----
HsLRRK1_RC  LPVANDSYLLPHLLPSKPGLDTHGMRHPTANTIQRVFKMSFVPVGFWQRFIARMLISLAE
            **+  +  *+*  *       +         +  *  *+++* +  *+***  * *  *+*

                   430        440        450        460        470        480
HsLRRK2_RC  -----------------EISPYMLSGRER-------ALRPNRMYWRQGIYLNWSPEAYCL
HsLRRK1_RC  MDLQLFENKKNTKSRNRKVTIYSFTGNQRNRCSTFRVKRNQTIYWQEGLLVTFDGGYLSV
                             +++ *  +* +*             *   +**++*+ + +      +

                   490        500        510        520        530        540
HsLRRK2_RC  VGSEVLDNHPES-FLKITVPSCRKGCILLGQVVDHIDSLMEEWFPGLLEIDICGE-----
HsLRRK1_RC  ESSDVNWKKKKSGGMKIVCQSEVRDFSAMAFITDHVNSLIDQWFPALTATESDGTPLMEQ
              *+*      +*  +**    *  +     +  + **++**+++*** *   +   *

                   550        560        570        580
HsLRRK2_RC  ---GETLLKKWALYS--FNDGEEHQKILLDDLMKKAEEGDLLVNPD
HsLRRK1_RC  YVPCPVCETAWAQHTDPSEKSEDVQYFDMEDCVLTAIERDFISCPR
                      ** ++       *+ *    ++* +   *  *  *  +   *
```

Sequence alignment of the LRRK2 KIN domain (amino acids 1845 to 2139) with the LRRK1 KIN domain (amino acids 1208 to 1527)

Secondary structure elements (NetSurfP [1]) are indicated: α-helices are red *italic*, and β-strands yellow underlined. LRRK2 pathogenic (G2019S and I2020T) and functional (K1906M (conserved K in β3) and D1994A (part of the catalytic loop)) mutations are boxed. Putative phosphorylation sites[#1] are double underlined. The conserved motifs are highlighted in **bold** and labelled. The KIN domains share 30 % (101 / 328) sequence identity and 50 % (165 / 328) sequence similarity. Identical residues are marked with an asterisk (*) and similar residues are marked with a plus sign (+).

```
                      10        20        30        40        50        60
                                                        P-loop
HsLRRK2_KIN   QPRLTIPISQIAPDLILADLPRNIMLNNDELEFEQAPEFLLGDGSFGSV-YRAAYEGEEV
HsLRRK1_KIN   HPDLPVPLQELVPELFMTDFPARLFLENSKLEHSEDEGSVLGQGGSGTVIYRARYQGQPV
               * * +*+ ++ *+* + * *  + * * +**  +    +** *  *+* *** *+*+ *

                      70        80        90       100       110       120
                K (β3)                                E (αC)
HsLRRK2_KIN   AVKIFN-----------KHTSLRLLR------------QELVVLCHLHHPSLISLLAAGI
HsLRRK1_KIN   AVKRFHIKKFKNFANVPADTMLRHLRATDAMKNFSEFRQEASMLHALQHPCIVALIGISI
              *** *+             * ** **            **  +*  * ** +++*+   *

                     130       140       150       160       170       180
                   gk                                                  cata-
HsLRRK2_KIN   RPRMLVMELASKGSLDRLLQQDKAS-----LTRTLQHRIALHVADGLRYLHSAMIIYRDL
HsLRRK1_KIN   HPLCFALELAPLSSLNTVLSENARDSSFIPLGHMLTQKIAYQIASGLAYLHKKNIIFCDL
               *    +***   **+ +* ++        *   *  +**  +* ** ***   **+ **

                     190       200       210       220       230       240
              lytic loop                 activation loop
HsLRRK2_KIN   KPHNVLLFTLYPNAAIIAKIADYGIAQYCCRMGIKTSEGTPGFRAPEVARGNVIYNQQAD
HsLRRK1_KIN   KSDNILVWSLDVKEHINIKLSDYGISRQSFHEGALGVEGTPGYQAPEI-RPRIVYDEKVD
              *  *+*+++*     *  *++****++     *    *****++***+ *  ++*+++ *

                     250       260       270       280       290       300
HsLRRK2_KIN   VYSFGLLLYDILTTGGRIVEGLKFPNEFDELEIQGKLPDPVKEYGCAP----WPMVEKLI
HsLRRK1_KIN   MFSYGMVLYELLS-GQRPALG------HHQLQIAKKLSKGIRPVLGQPEEVQFRRLQALM
              ++*+*++**++*+ * *   *        +*+*  **   ++     *    +  ++ *+

                     310       320
HsLRRK2_KIN   KQCLKENPQERPTSAQVFDILNSAELVC
HsLRRK1_KIN   MECWDTKPEKRPLALSVVSQMKDPTFAT
               +*    *++** +  *   +
```

#1) T1849 [7], S1853 [7], T1912 [7], S1913 [7], T1967 [7,11], T1969 [7,11], T2031 [6,7,12], S2032 [6,12], T2035 [7,12]

Sequence alignment of the LRRK2 WD40 repeat domain (amino acids 2140 to end) with the LRRK1 C-terminal domain (amino acids 1528 to end)

Secondary structure elements (NetSurfP [1]) are indicated: α-helices are red *italic*, and β-strands yellow underlined. LRRK2 risk factor (G2385R) is boxed. Putative phosphorylation sites[#1] are double underlined. The identity and similarity scores are 12 % (61 / 501) and 27 % (136 / 501) respectively. Identical residues are marked with an asterisk (*) and similar residues are marked with a plus sign (+).

```
                    10        20        30        40        50        60
HsLRRK2_WD  LTRRILLPKNVIVECMVATHHNSRNASIWLGCGHTDRGQLSFLDLNTEGYTSEEVADSRI
HsLRRK1_WD  -MYELCCGKQ---TAFFSSQGQEYTVVFWDG----KEESRNYTVVNTEK---GLMEVQRM
                +   *        ++         * *         ++  +***      +   *+

                    70        80        90       100       110       120
HsLRRK2_WD  LCLALVHLP-VEKESWIVSGTQSGTLLVINTEDGKKRHTLEKMTDSVTCLYCNSFSKQSK
HsLRRK1_WD  CCPGMKVSCQLQVQRSLWTATEDQKIYIYTLKGMCPLNTPQQALDTPAVVTCFLAVPVIK
             *  +      + +  + + *+   + +   +     +* ++  *+   + *       *

                   130       140       150       160       170       180
HsLRRK2_WD  QKNFL-LVGTADGKLAIFEDKTVKLKGAAPLKILNIGNVSTPLMCLSESTNS--TERNVM
HsLRRK1_WD  KNSYLVLAGLADGLVAVFPVVRGTPKDSCSYLCSHTANRSKFSIADEDARQNPYPVKAME
            + ++* * * *** +*+*       * +      +  * *   +   ++  +    + +

                   190       200       210       220       230       240
HsLRRK2_WD  WGGCGTKIFSFSNDFTIQKLIETRTSQLFSYAAFSDSNIITVVV-------DTALYIAKQ
HsLRRK1_WD  VVNSGSEVW-YSNGPGLLVIDCASLEICRRLEPYMAPSMVTSVVCSSEGRGEEVVWCLDD
                *++++ +**   +  +             +   +++* **       +  ++

                   250       260       270       280       290       300
HsLRRK2_WD  NSPVVEVWDKKTEKLCGLIDC--VHFLREVM-VKENKESKHKMSYSGRVKTLCLQK----
HsLRRK1_WD  KANSLVMYHSTTYQLCARYFCGVPSPLRDMFPVRPLDTEPPAASHTANPKVPEGDSIADV
             +  + ++   * +**    *     **++  *+         *++   *

                   310       320       330       340       350       360
HsLRRK2_WD  ------------------------------------------------------------
HsLRRK1_WD  SIMYSEELGTQILIHQESLTDYCSMSSYSSSPPRQAARSPSSLPSSPASSSSVPFSTDCE

                   370       380       390       400       410       420
HsLRRK2_WD  ------------------NTALWIGTGGGHILLLD-LSTRRLIRVIYNFCNSVRVMMTAQ
HsLRRK1_WD  DSDMLHTPGAASDRSEHDLTPMDGETFSQHLQAVKILAVRDLIWVPRRGGDVIVIGLEKD
                               * +   *   *+  +  *+ * ** *     + + + +

                   430       440       450       460       470       480
HsLRRK2_WD  LGSLK-NVMLVLGYNRKNTEG---------------TQKQKEIQSCLTVWDINLPHEVQN
HsLRRK1_WD  SGAQRGRVIAVLKARELTPHGVLVDAAVVAKDTVVCTFENENTEWCLAVWRGWGAREFDI
             *+ +  *+ **        *               * + +  + **  **      *

                   490
HsLRRK2_WD  LEKHIEVRKELAEKMRRTSVE
HsLRRK1_WD  FYQSYEELGRLEACTRKRR--
              +  *    *    *+
```

[#1]) S2257 [7], T2483 [2], T2524 [7]

Sequence alignment of the WD40 domain repeats of LRRK2

We could detect the presence of 7 putative WD40 repeats in the C-terminal domain of LRRK2 **(amino acid 2143 to 2498)**. No WD40 repeats were detected in the LRRK1 C-terminal sequence. Also, no other known protein fold was recognized when using the fold recognition server Phyre [13]. At the same time, the WD40 fold was positively detected for the LRRK2 C-terminal domain via Phyre. The consensus sequence was calculated such that residues occurring in (more than) 50 % of the repeats are given. Residue conservation is coloured as explained in the consensus (C) sequence (X = any residue, ϕ = hydrophobic residue (A, F, I, L, M, V, W; blue), + = charged residue (D, E, H, K, R; violet), $ = polar, non-charged residue (N, Q, S, T, Y; orange), £ = special amino acids (P, C, G; brown)), - is a possible insertion or deletion site and var is a variable region. The risk factor in the LRRK2 WD40 domain reported is highlighted in red (G2385R).

LRRK2 WD40 REPEATS

**7a**																									R	I	L	L	PKNV
**1**	IVE	C	M	V	A	T	H	HNSRNAS	I	W	L	G	C	G	H	TDR	G	Q	L	S	F	L	D	LNTEG	Y	T	S	E	EVAD
**2**	SRIL	C	L	A	L	V	H	LPVEKE	S	W	I	V	S	G	T	QS	G	T	L	L	V	I	N	TEDGKKR	H	T	L	E	KMTD
**3**	SVT	C	L	Y	C	N	S	FSKQSKQK	N	F	L	L	V	G	T	AD	G	K	L	A	I	F	E	DKTVKLKGAAPL	K	I	L	N	IGNV
**4**	STPLM	C	L	S	E	S	T	NSTERNV	M	W	G	G	C	G	T	-	K	I	F	S	F	S	N	DFTIQ	K	L	I	E	TRTSQLFSYAAF
**5**	SDS	N	I	I	T	V	V	VDTA	L	Y	I	A	K	Q	N	S	P	V	V	E	V	W	D	KKTEKLC	G	L	I	D	CVHF
**6**	LRE	V	M	V	K	E	N	**a**	L	W	I	G	T	G	G	-	G	H	I	L	L	L	D	LSTR	R	L	I	R	VIYNFCN
**7b**	SVR	V	M	M	T	A	Q	LGSLKN	V	M	L	V	L	G	Y	**b**	S	C	L	T	V	W	D						
**C**	**var**	**C**	**ϕ**	**ϕ**	**X**	**X**	**$**	**var**	**ϕ**	**W**	**ϕ**	**ϕ**	**X**	**G**	**$**	**var**	**G**	**X**	**L**	**X**	**ϕ**	**ϕ**	**D**	**var**	**+**	**ϕ**	**ϕ**	**+**	**var**

a: KESKHKMSYSGRVKTLCLQKNTA; **b:** NRKNTEGTQKQKEIQ

Supplementary Figure Legends

Figure S1: Binding Buffers optimization by ProteoPlex. ProteoPlex melting curve profiles obtained for LRRK2 bound to affinity beads in the presence of 88 different buffers (the binding buffer screen). A standard ProteoPlex 88-buffer screen was performed to find the buffer system in which affinity-bound 3xflag-LRRK2 was most stable. The starting material for the screen was 3-flagLRRK2 that had been purified in Tris pH 7.5 based lysis and wash buffers and stored at 4ºC (while still bound to the affinity beads) in storage buffer as described in *Materials and Methods*. At the moment of testing, affinity beads loaded with protein were rinsed with Tris pH 7.5 based buffer as described in *Materials and Methods* and used immediately afterwards. The melting curves shown are for the best condition calculated by ProteoPlex, Hepes pH 6.8 (green), the initial buffer, Tris pH 7.5 (red), and the worst condition, MES pH 5.5 (blue). Hepes pH 6.8 was used to adapt lysis and wash buffers for all subsequent protein purifications (see *Results* sections for exact compositions).

Figure S2 : Additive optimization by ProteoPlex screening. A standard ProteoPlex 88-ligand screen was performed to find additives that enhance the stability of 3xflag-LRRK2 when this is bound to the anti-flag affinity beads. The starting material for the screen was 3-flagLRRK2 that had been purified as described in *Materials and Methods*, with the replacement of Tris pH 7.5 by Hepes pH 6.8 in lysis, wash, storage and rinse buffers. Affinity beads loaded with protein was stored at 4ºC (while still bound to the affinity beads) in storage buffer with Hepes pH 6.8 replacing Tris pH 7.5. The beads were rinsed as described in *Materials and Methods* (with the adaptation of the buffer to replace Tris pH 7.5 with Hepes pH 6.8) and used immediately afterwards. The resulting melting curve profiles are shown. The curves were assessed by visual inspection. Protein stability relates to an exponential increase / exponential decrease (cooperativity) of the curves. The most favorable melting curves are enclosed in boxes. (See *Materials and Methods* and *Results* sections for further details). As a result, the composition of the lysis/binding buffer used to purify 3-flagLRRK2 for the cryo-EM experiments was adapted to 20 mM Hepes pH 6.8, 10 mM CaCl2, 5 mM MgCl2, 100 mM NH4Cl, 1% (v/v) Triton, 5% (v/v) glycerol.

Figure S3. Elution buffer optimization by ProteoPlex screening. The stability of 3xflag-LRRK2 in 24 different elution buffers was assessed. The starting material for the screen was 3-flag LRRK2 that had been purified in Hepes pH 6.8 based buffer (see *Results* sections for more details) and stored at 4ºC (while still bound to the affinity beads) in the same buffer supplemented with 50% glycerol. The beads were rinsed with the elution buffers to be tested and protein was eluted and tested as described in *Materials and Methods*. The resulting melting curve profiles are shown. The curves were assessed by visual inspection. Protein stability relates to an exponential increase / exponential decrease (cooperativity) in the melting curves via the visual inspection.

The most favorable melting curves are enclosed in boxes. As a result, Hepes pH 8.2 was selected and used as the elution buffer when 3xflag-LRRK2 was purified for cryo-EM. Based on this result, the composition of the elution buffer for 3xflag-LRRK2 was adapted to 20 mM Hepes pH 8.2, 100 mM NH4Cl, 5 mM MgCl2, 10 mM CaCl2 and Triton 0,02%.

Figure S4. Vitrification of LRRK2 using holey grids by cryo-EM. LRRK2 sample as imaged in a holey grid. All the LRRK2 particles (shown by black arrows) are sticking to the carbon on the edges of the holes and none in the vitrified ice. Scale bar: 100 nm.

Figure S5. Reference free class averaging of LRRK2 in EMAN2 and RELION. The total data set contained 15'352 LRRK2 particles. **A.** The 128 reference free class averages obtained after 5 iterations in EMAN2 using e2refine2d program. The best classes from these are shown in Figure 5 A and subsequently used to generate and refine the initial 3D model for LRRK2. **B.** The 128 reference free class averages obtained after 25 iterations in RELION. The class averages show structural heterogeneity among particles. **Below:** Selected examples. The shape and dimensions of the particles are very similar to that obtained with EMAN2. Scale bar, 10nm.

Figure S6. Resolution curves for the LRRK2 and LRRK1 3D models. A. The resolutions of the 3xflag-LRRK2 map is 24.2 Å using the gold standard 0.143 FSC cutoff criteria. **B**. The resolutions of the 3xflag-LRRK1 map is 24.3 Å using the gold standard 0.143 FSC cutoff criteria. In both A and B, arrows point to the calculated resolution of the 3D models.

Figure S7. Spectral signal-to-noise ratio curve (SSNR) for the LRRK2. The SSNR of best class crossed 1.0 at 16.2 Angstroms resolution, as indicated by the arrow. The 3D model of LRRK2 representing the best class is shown in Figure 7.

Figure S1:

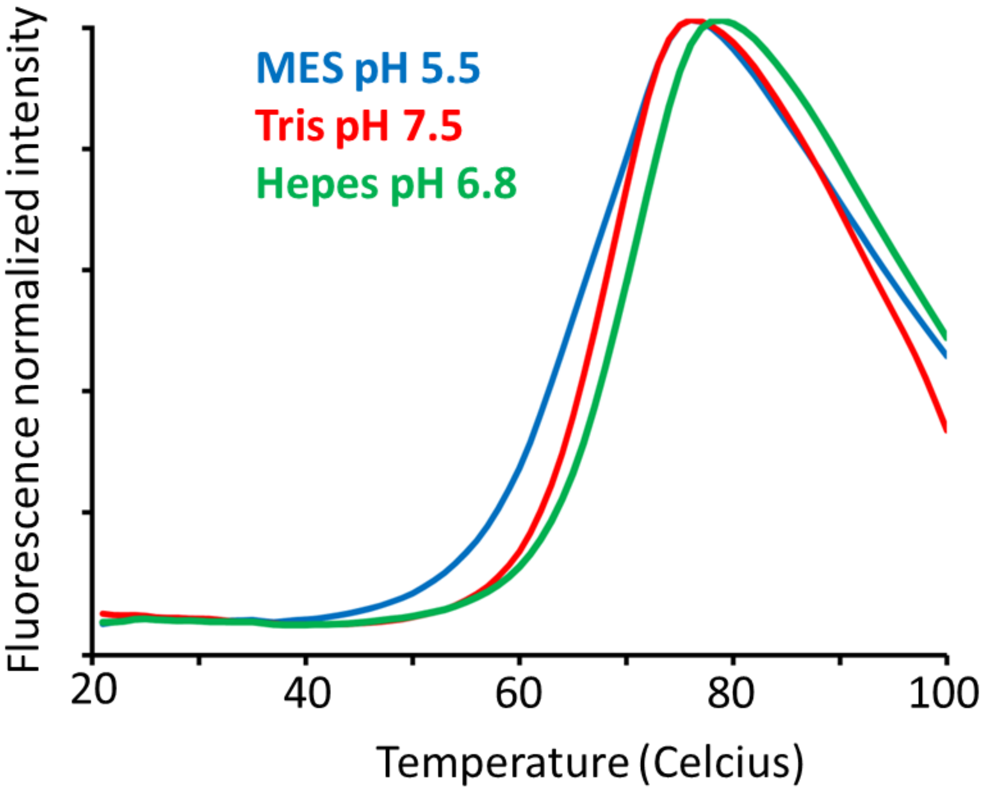
MES pH 5.5
Tris pH 7.5
Hepes pH 6.8
Fluorescence normalized intensity
20
40
60
80
100
Temperature (Celcius)

Figure S2:

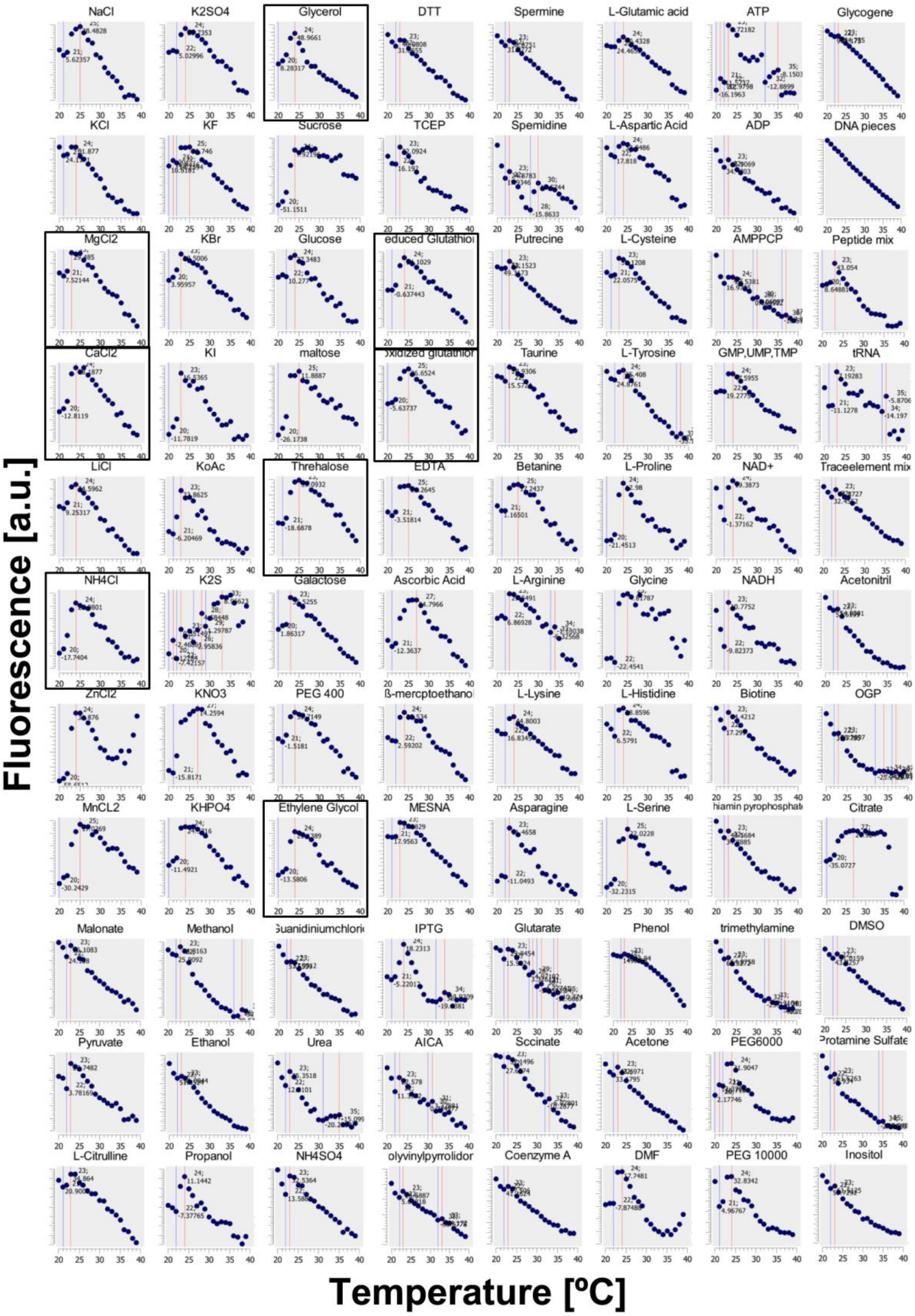
NaCl
K2SO4
Glycerol
DTT
Spermine
L-Glutamic acid
ATP
Glycogene
KCl
KF
Sucrose
TCEP
Spemidine
L-Aspartic Acid
ADP
DNA pieces
MgCl2
KBr
Glucose
Putrecine
L-Cysteine
AMPPCP
Peptide mix
CaCl2
KI
maltose
Taurine
L-Tyrosine
GMP,UMP,TMP
tRNA
LiCl
KoAc
Threhalose
EDTA
Betanine
L-Proline
NAD+
NH4Cl
K2S
Galactose
Ascorbic Acid
L-Arginine
Glycine
NADH
Acetonitril
ZnCl2
KNO3
PEG 400
L-Lysine
L-Histidine
Biotine
OGP
MnCL2
KHPO4
Ethylene Glycol
MESNA
Asparagine
L-Serine
Citrate
Malonate
Methanol
IPTG
Glutarate
Phenol
trimethylamine
DMSO
Pyruvate
Ethanol
Urea
AICA
Sccinate
Acetone
PEG6000
L-Citrulline
Propanol
NH4SO4
Coenzyme A
DMF
PEG 10000
Inositol
Fluorescence [a.u.]
Temperature [ºC]

Figure S3:

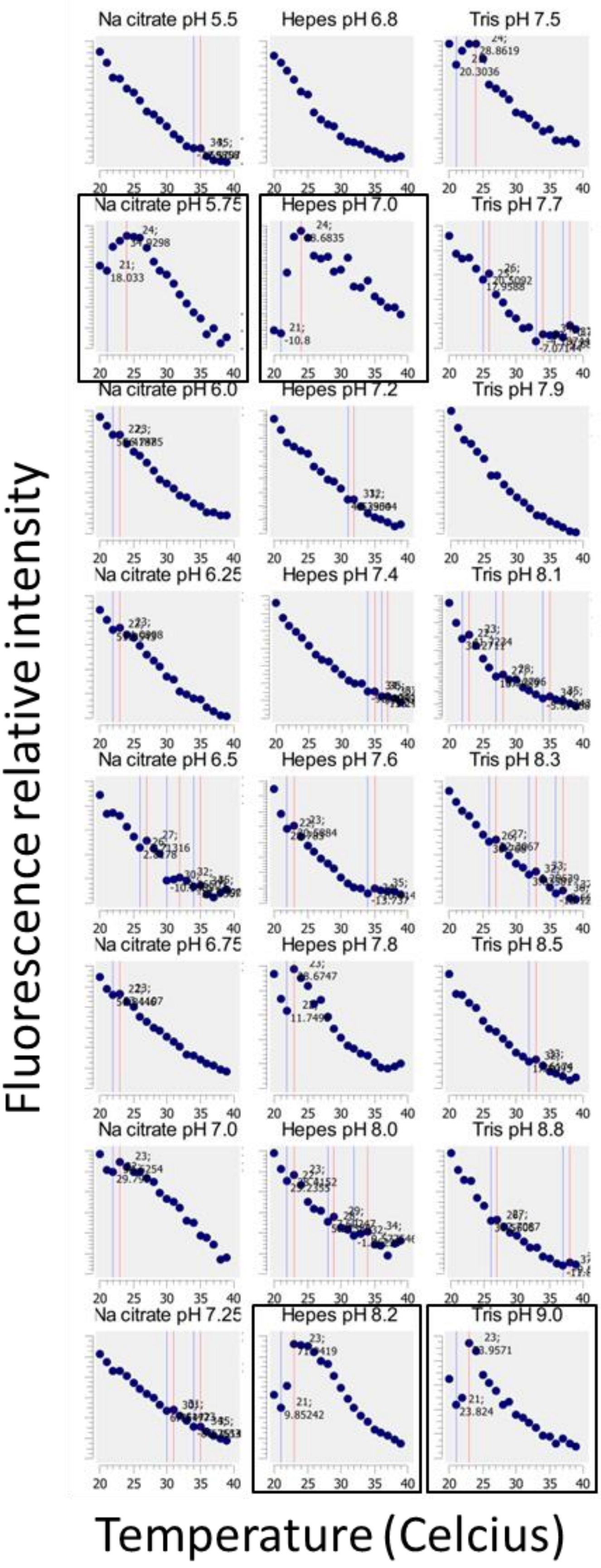
Na citrate pH 5.5
Hepes pH 6.8
Tris pH 7.5
Na citrate pH 5.75
Hepes pH 7.0
Tris pH 7.7
Na citrate pH 6.0
Hepes pH 7.2
Tris pH 7.9
Na citrate pH 6.25
Hepes pH 7.4
Tris pH 8.1
Na citrate pH 6.5
Hepes pH 7.6
Tris pH 8.3
Na citrate pH 6.75
Hepes pH 7.8
Tris pH 8.5
Na citrate pH 7.0
Hepes pH 8.0
Tris pH 8.8
Na citrate pH 7.25
Hepes pH 8.2
Tris pH 9.0
Fluorescence relative intensity
Temperature (Celcius)

Figure S4:

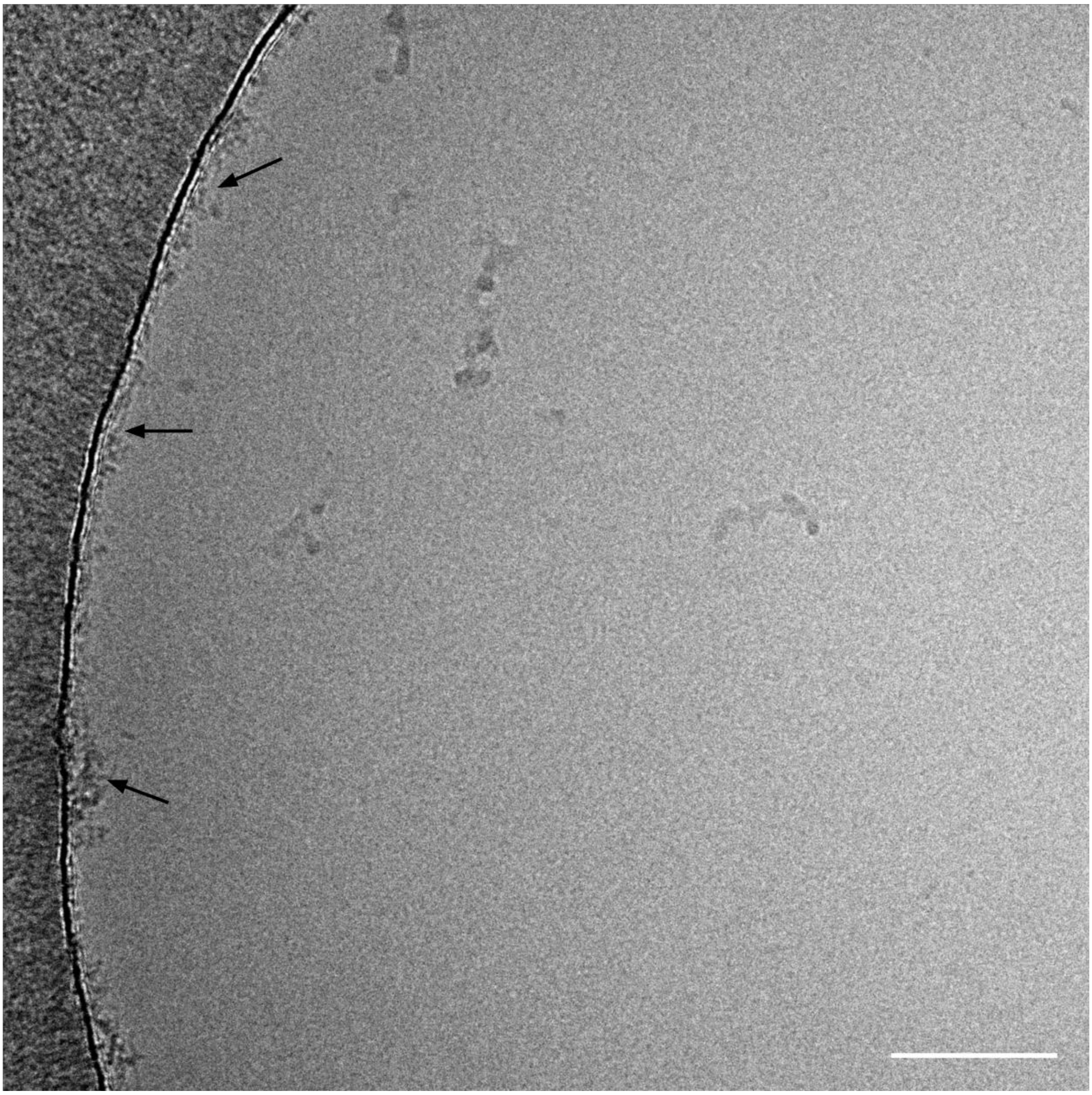

Figure S5:

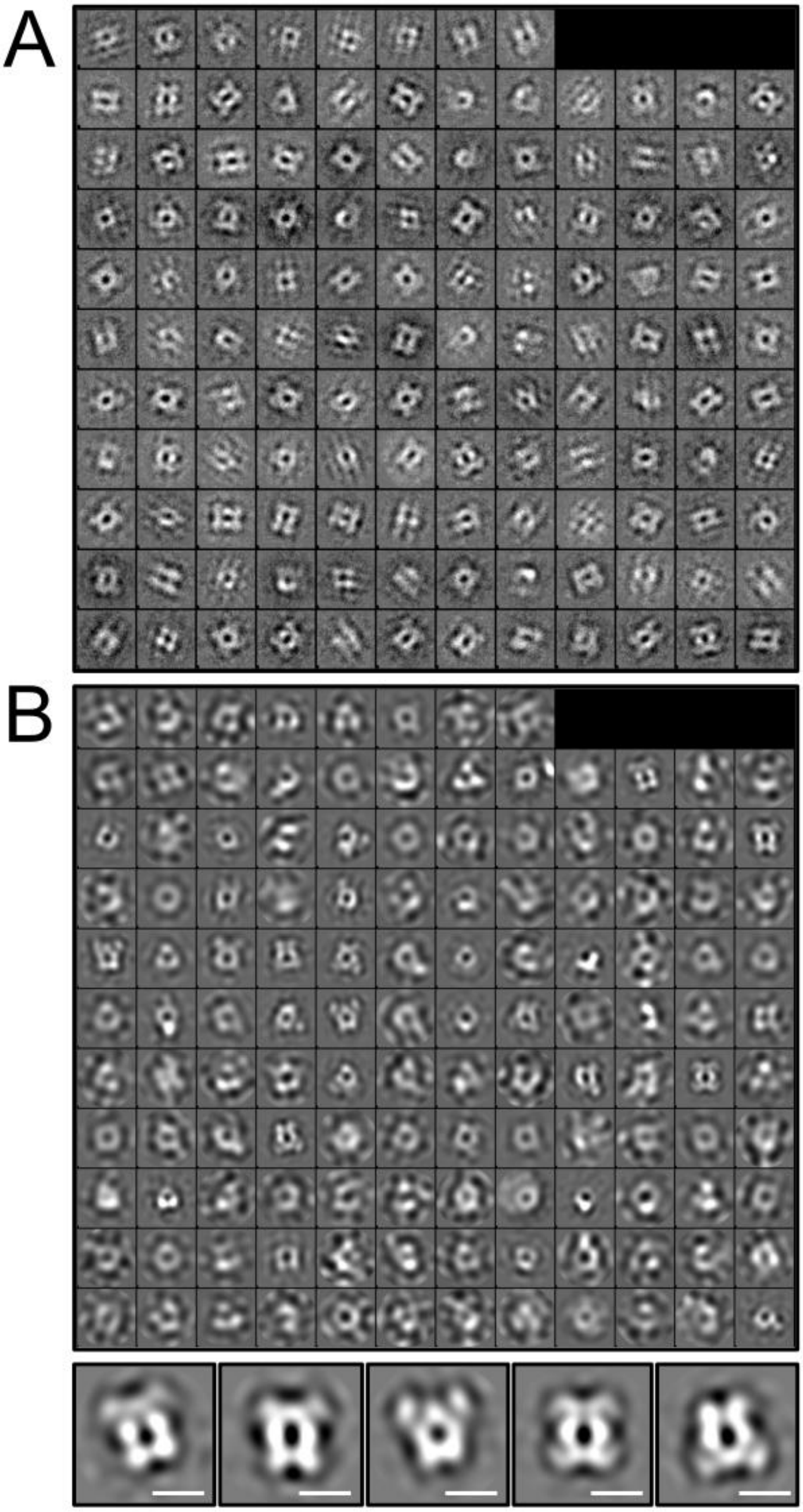

Figure S6:

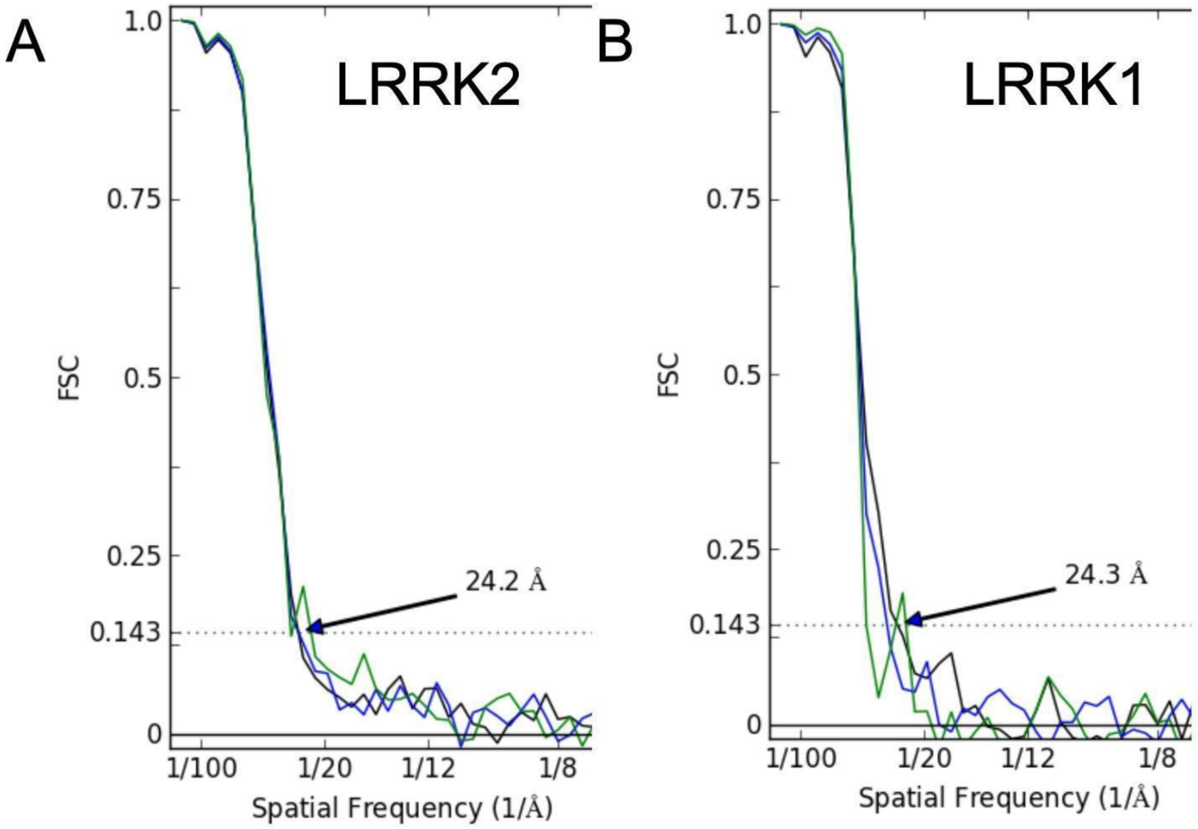
A
LRRK2
FSC
1.0
0.75
0.5
0.25
0.143
0
24.2 Å
1/100
1/20
1/12
1/8
Spatial Frequency (1/Å)
B
LRRK1
FSC
1.0
0.75
0.5
0.25
0.143
0
24.3 Å
1/100
1/20
1/12
1/8
Spatial Frequency (1/Å)

Figure S7:

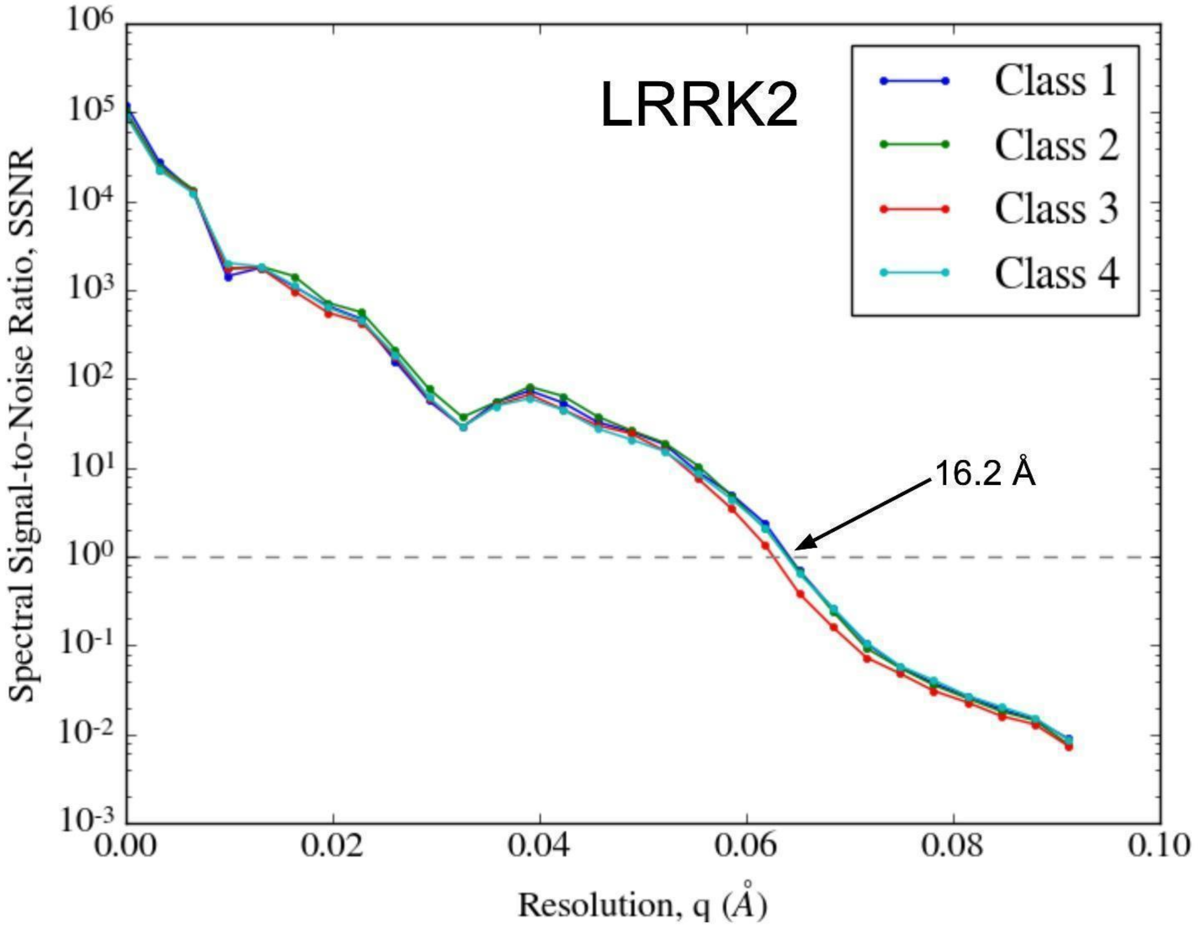
LRRK2
Class 1
Class 2
Class 3
Class 4
16.2 Å
Spectral Signal-to-Noise Ratio, SSNR
Resolution, q (Å)
10^6
10^5
10^4
10^3
10^2
10^1
10^0
10^-1
10^-2
10^-3
0.00
0.02
0.04
0.06
0.08
0.10

Bibliography

1 Petersen, B., Petersen, T. N., Andersen, P., Nielsen, M. & Lundegaard, C. A generic method for assignment of reliability scores applied to solvent accessibility predictions. *BMC Struct Biol* **9**, 51, doi:1472-6807-9-51 [pii], doi:0.1186/1472-6807-9-51 (2009).

2 Gloeckner, C. J. *et al.* Phosphopeptide analysis reveals two discrete clusters of phosphorylation in the N-terminus and the Roc domain of the Parkinson-disease associated protein kinase LRRK2. *J Proteome Res* **9**, 1738-1745, doi:10.1021/pr9008578 (2010).

3 Nichols, R. J. *et al.* 14-3-3 binding to LRRK2 is disrupted by multiple Parkinson's disease-associated mutations and regulates cytoplasmic localization. *Biochem J* **430**, 393-404, doi:BJ20100483 [pii], doi:10.1042/BJ20100483 (2010).

4 Li, X. *et al.* Phosphorylation-dependent 14-3-3 binding to LRRK2 is impaired by common mutations of familial Parkinson's disease. *PLoS One* **6**, e17153, doi:10.1371/journal.pone.0017153 (2011).

5 Civiero, L. *et al.* Biochemical characterization of leucine rich reeat kinases 1 and 2 demonstrates formation of dimers in highly purified protein. *PloS One* (Submitted).

6 Greggio, E. *et al.* The Parkinson's disease kinase LRRK2 autophosphorylates its GTPase domain at multiple sites. *Biochem Biophys Res Commun* **389**, 449-454, doi:S0006-291X(09)01767-7 [pii], doi10.1016/j.bbrc.2009.08.163 (2009).

7 Pungaliya, P. P. *et al.* Identification and characterization of a leucine-rich repeat kinase 2 (LRRK2) consensus phosphorylation motif. *PLoS One* **5**, e13672, doi:10.1371/journal.pone.0013672 (2010).

8 Bosgraaf, L. & Van Haastert, P. J. Roc, a Ras/GTPase domain in complex proteins. *Biochim Biophys Acta* **1643**, 5-10, doi:S0167488903001435 [pii] (2003).

9 Gotthardt, K., Weyand, M., Kortholt, A., Van Haastert, P. J. & Wittinghofer, A. Structure of the Roc-COR domain tandem of C. tepidum, a prokaryotic homologue of the human LRRK2 Parkinson kinase. *EMBO J* **27**, 2352, doi:emboj2008167 [pii], doi:10.1038/emboj.2008.167 (2008).

10 Webber, P. J. *et al.* Autophosphorylation in the leucine-rich repeat kinase 2 (LRRK2) GTPase domain modifies kinase and GTP-binding activities. *J Mol Biol* **412**, 94-110, doi:S0022-2836(11)00798-4 [pii], doi:10.1016/j.jmb.2011.07.033 (2011).

11 Kamikawaji, S., Ito, G. & Iwatsubo, T. Identification of the autophosphorylation sites of LRRK2. *Biochemistry* **48**, 10963-10975, doi:10.1021/bi9011379 (2009).

12 Li, X., Moore, D. J., Xiong, Y., Dawson, T. M. & Dawson, V. L. Reevaluation of phosphorylation sites in the Parkinson disease-associated leucine-rich repeat kinase 2. *J Biol Chem* **285**, 29569-29576, doi:M110.127639 [pii], doi:10.1074/jbc.M110.127639 (2010).

13 Kelley, L. A. & Sternberg, M. J. Protein structure prediction on the Web: a case study using the Phyre server. *Nat Protoc* **4**, 363-371, doi:nprot.2009.2 [pii], doi:10.1038/nprot.2009.2 (2009).
